# Facile synthesis of AIEgens with wide color tunability for cellular imaging and therapy†
†Electronic supplementary information (ESI) available: CCDC 1886931 and 1886933. For ESI and crystallographic data in CIF or other electronic format see DOI: 10.1039/c8sc05805a


**DOI:** 10.1039/c8sc05805a

**Published:** 2019-02-22

**Authors:** Wenhan Xu, Michelle M. S. Lee, Zhihan Zhang, Herman H. Y. Sung, Ian D. Williams, Ryan T. K. Kwok, Jacky W. Y. Lam, Dong Wang, Ben Zhong Tang

**Affiliations:** a Center for AIE Research , College of Materials Science and Engineering , Shenzhen University , Shenzhen 518060 , China . Email: wangd@szu.edu.cn; b Hong Kong Branch of Chinese National Engineering Research Center for Tissue Restoration and Reconstruction , Department of Chemistry , Institute of Molecular Functional Materials , State Key Laboratory of Neuroscience , Division of Biomedical Engineering , Division of Life Science , The Hong Kong University of Science and Technology , Clear Water Bay , Kowloon , Hong Kong , China . Email: tangbenz@ust.hk

## Abstract

Luminogens with aggregation-induced emission (AIE) characteristics are nowadays undergoing explosive development in the fields of imaging, process visualization, diagnosis and therapy. However, exploration of an AIE luminogen (AIEgen) system allowing for extremely wide color tunability remains challenging. In this contribution, the facile synthesis of triphenylamine (TPA)–thiophene building block-based AIEgens having tunable maximum emission wavelengths covering violet, blue, green, yellow, orange, red, deep red and NIR regions is reported. The obtained AIEgens can be utilized as extraordinary fluorescent probes for lipid droplet (LD)-specific cell imaging and cell fusion assessment, showing excellent image contrast to the cell background and high photostability, as well as satisfactory visualization outcomes. Interestingly, quantitative evaluation of the phototherapy effect demonstrates that one of these presented AIEgens, namely TTNIR, performs well as a photosensitizer for photodynamic ablation of cancer cells upon white light irradiation. This study thus provides useful insights into rational design of fluorescence systems for widely tuning emission colors with high brightness, and remarkably extends the applications of AIEgens.

## Abstract 2

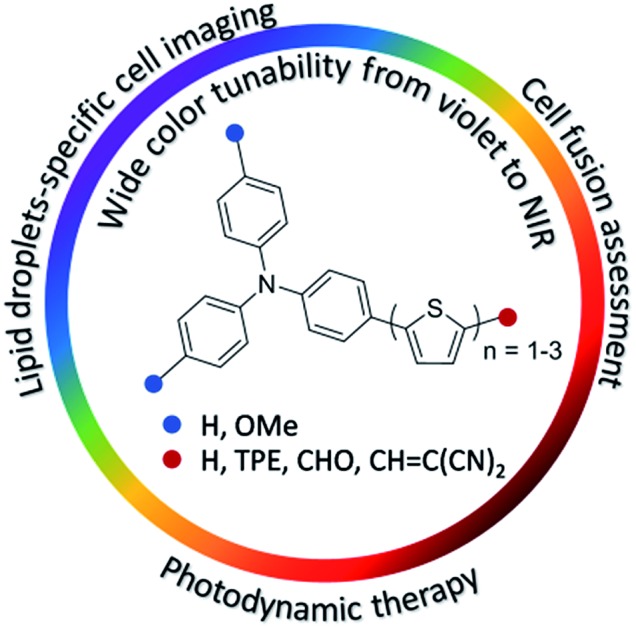
Facile synthesis and bio-applications of a series of AIEgens with widely tunable emissions ranging from violet to near-infrared are reported.

## Introduction

The exploration of fluorescent materials and technologies has opened new avenues to scientific advancement, societal development and public health,^1^ which is exemplified by the Nobel Prize successively awarded to fluorescence-related research. As one of the most important branches of fluorescent materials, fluorescent bio-materials that offer researchers a powerful platform for analytical sensing and optical imaging have been proven to be extremely useful for biological visualization, clinical diagnosis and disease treatment by virtue of their non-invasiveness, *in situ* workability, excellent accuracy, superb sensitivity and simple operation.^2^ Although many types of fluorophores have been commercialized for biological applications, the current situation is still far from ideal, mainly due to some limitations: (1) inherent fluorescence quenching upon aggregate formation due to intermolecular π–π stacking and other nonradiative pathways, which is notoriously known as aggregation-caused quenching (ACQ);^3^ (2) the difficulty of widely tuning emission colors by simple modification of molecular structures; and (3) complicated and laborious syntheses of fluorophores.^4^

As an anti-ACQ phenomenon, aggregation-induced emission (AIE) was coined in 2001 by Tang's group,^5^ which refers to a unique phenomenon that a novel class of fluorophores are non-emissive or weakly emissive in the molecularly dissolved state but they emit intensively in aggregated states owing to the restriction of the intramolecular motions (RIMs).^6^ Remarkably, the AIE principle has triggered state-of-the-art developments in an array of biological fields, ranging from bioimaging, biosensing, stimuli–responsive systems, and therapeutics to theranostics, mainly resulting from various impressive advantages of AIE luminogens (AIEgens), such as high photobleaching threshold, high signal-to-noise ratio for imaging, excellent tolerance for any concentrations, large Stokes shift, turn-on feature for detecting analytes, and efficient photosensitizing ability.^7^ Although numerous AIEgens have been constructed on the basis of different structural motifs including tetraphenylethene,^8^ hexaphenylsilole,^9^ tetraphenylpyrazine^10^ and distyrylanthracene,^11^ to the best of our knowledge, there has been no single AIE system which allows arbitrarily tuning emissions ranging from each color of visible light to the near-infrared (NIR) region. Considering the great significance of tunable fluorescent systems in the applications of multi-target sensing, optoelectronic devices and full-color bio-imaging,^12^ the development of an AIE system exhibiting wide color tunability is highly desired and remains a challenging task.

**Fig. 1:**
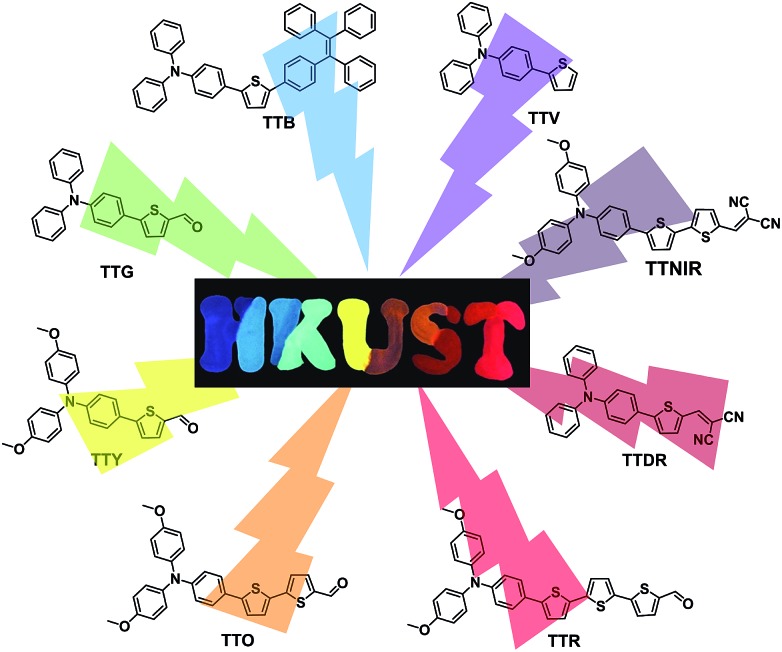
TPA–thiophene-based AIEgens with widely tunable emissions.

Compared with inorganic complexes and quantum dots, organic fluorophores are advantageous for bio-imaging, diagnosis and therapy, benefiting from their good bio-compatibility, tunable molecular structures and chemical compositions, and scalable synthesis.^13^ Evidently, the exploration of an organic fluorophore system with both the AIE attribute and emission color tunability across a wide wavelength range would captivate much interest. Herein, we report for the first time the design and synthesis of a series of AIEgens having widely tunable emissions covering violet, blue, green, yellow, orange, red, deep red and NIR regions (Fig. 1). Each AIEgen comprising the triphenylamine (TPA)–thiophene building block is facilely obtained through one- or two-step reaction, and the emission colors are tuned by simple alteration of the HOMO–LUMO energy level by the introduction of electron donor (D)–acceptor (A) substituents.^14^ Moreover, these AIEgens can be successfully utilized as extraordinary lipid droplet (LD)-specific bioprobes in cell imaging, determination of cell fusion, and photodynamic cancer cell ablation.

## Results and discussion

### Synthesis and single crystal analysis

**Scheme 1:**
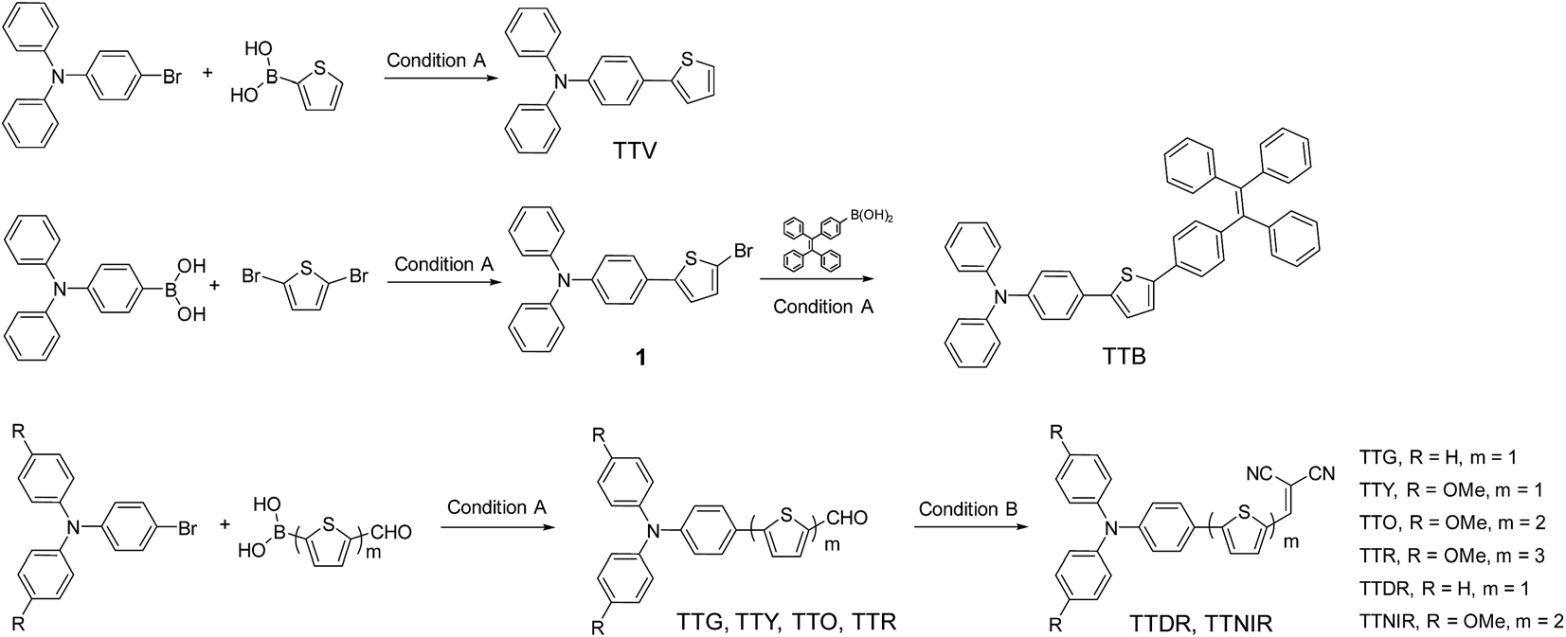
Synthetic routes to AIEgens with tunable emission colors. Condition A: Pd(PPh_3_)_4_, K_2_CO_3_, THF, H_2_O, 75 °C, 12 h. Condition B: EtOH, malononitrile, 78 °C, 72 h.

As depicted in Scheme 1, the desired compounds were facilely prepared through one or two steps. TTV was synthesized through the Suzuki–Miyaura coupling reaction of 4-bromo-*N*,*N*-diphenylaniline with thiophen-2-ylboronic acid in the presence of the palladium catalyst using mixed THF/H_2_O as the solvent at 75 °C. The same synthetic procedure was successfully conducted by employing substituted 4-bromo-*N*,*N*-diphenylaniline and modified thiophen-2-ylboronic acid as starting materials, producing compounds TTG, TTY, TTO and TTR. The reactions between TTG/TTO and malononitrile proceeded smoothly, giving the corresponding products TTDR and TTNIR with moderate yields. In addition, TTB was obtained by the Suzuki–Miyaura coupling reaction of (4-(1,2,2-triphenylvinyl)phenyl)boronic acid with intermediate product **1**, which was isolated through the Suzuki–Miyaura coupling reaction between (4-(diphenylamino)phenyl)boronic acid and 2,5-dibromothiophene.

**Fig. 2:**
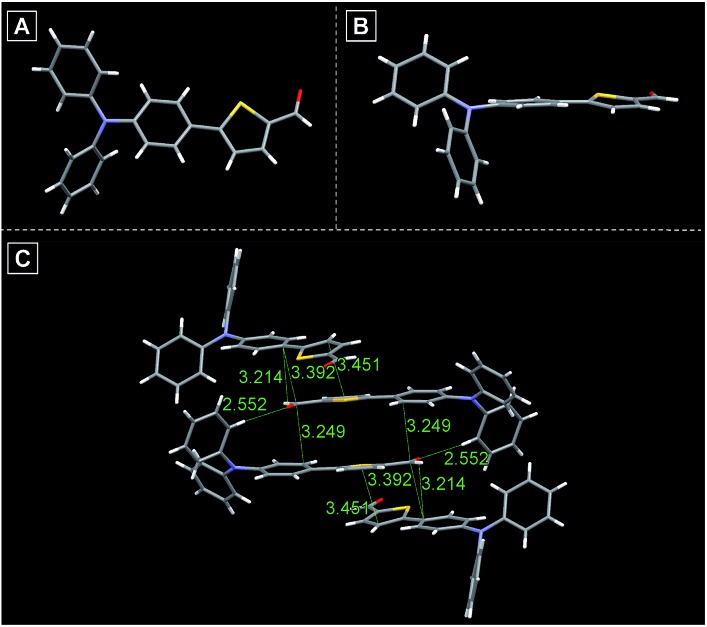
(A) Single crystal structure of TTG. (B) Side view of the crystal structure of TTG. (C) Various inter- and intramolecular interactions in crystals of TTG.

All compounds are composed of sufficient moieties that can freely rotate in the single-molecule state leading to energy consumption of the excited state through non-radiative pathways, thus ensuring that these compounds are weakly emissive in solution. Aiming to further study and deciphering their optical properties in the aggregation state, single crystals of TTG, TTY and TTDR were grown in DCM–MeOH mixtures by slow solvent evaporation. As illustrated in Fig. 2, S1 and S2,† the twisted conformation of the TPA segment extends the intermolecular distance (>3.2 Å) between two parallel planes, remarkably reducing or avoiding the intermolecular π–π interactions, and essentially preventing emission quenching in its aggregation state. Moreover, the molecular conformation can be strongly rigidified by abundant intermolecular interactions (such as C–H···O, C–H···C, and S···C) resulting in the restriction of molecular motions, which is beneficial for enhancing the solid state emission efficiency. On the basis of the above-mentioned XRD results, it is believed that these synthesized compounds are potentially AIE-active.

### Photophysical properties

**Fig. 3:**
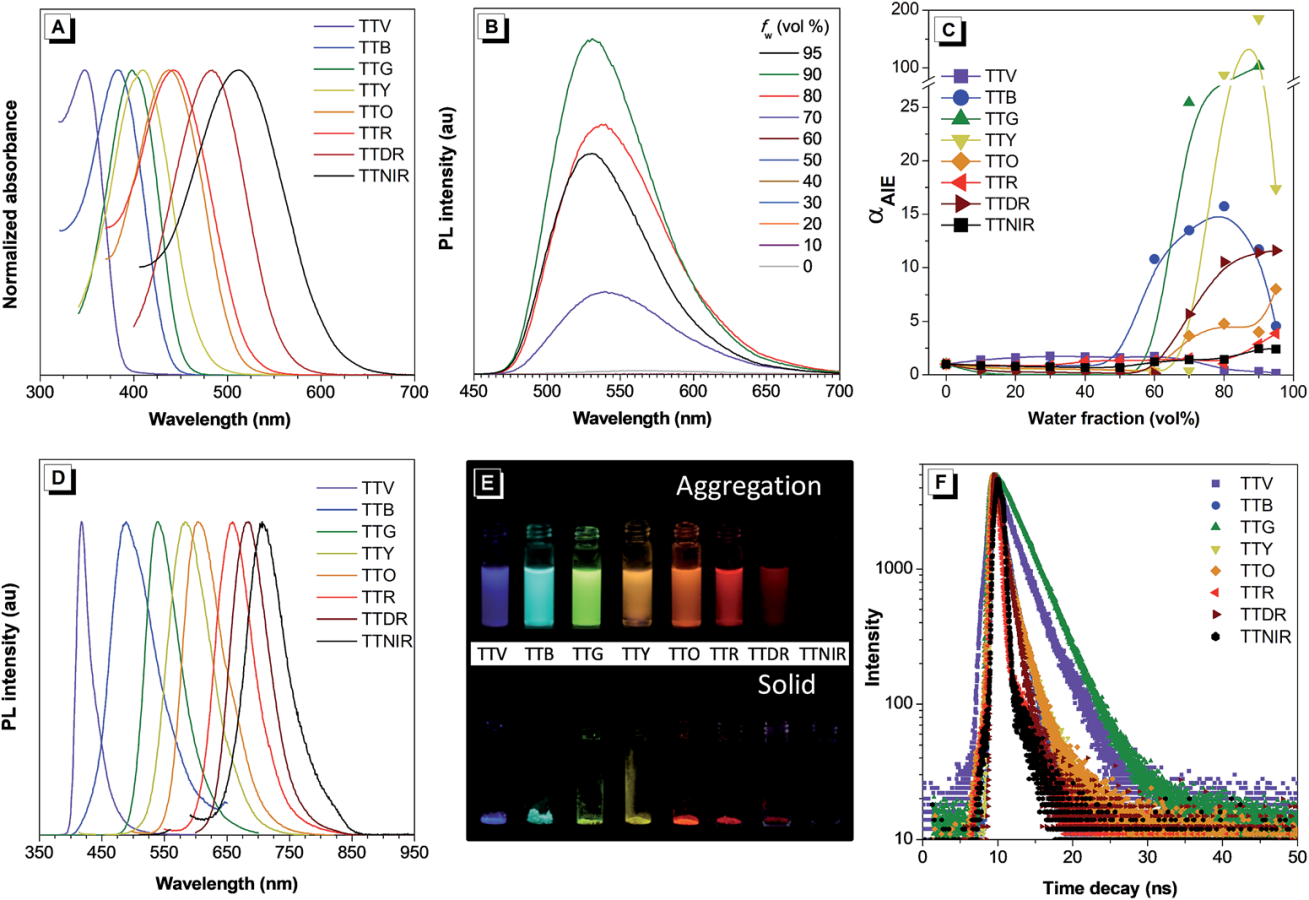
(A) Normalized absorption spectra of TTV, TTB, TTG, TTY, TTO, TTR, TTDR and TTNIR in ACN solution. (B) PL spectra of TTY (1 × 10^–5^ M) in ACN/water mixtures with different water fractions (*f*_w_); *λ*_ex_: 410 nm. (C) The plot of the emission maximum and the relative emission intensity (*I*/*I*_0_) *versus* the composition of the aqueous mixture of TTV, TTB, TTG, TTY, TTO, TTR, TTDR and TTNIR. (D) Normalized PL spectra of TTV (*λ*_em_: 417 nm), TTB (*λ*_em_: 489 nm), TTG (*λ*_em_: 539 nm), TTY (*λ*_em_: 583 nm), TTO (*λ*_em_: 603 nm), TTR (*λ*_em_: 659 nm), TTDR (*λ*_em_: 684 nm), and TTNIR (*λ*_em_: 706 nm) in the solid state. (E) Fluorescence photographs of TTV, TTB, TTG, TTY, TTO, TTR, TTDR and TTNIR (from left to right) in ACN/water mixtures with 95% water fractions (upper) and in the solid state (below) taken under 365 nm UV irradiation. (F) Fluorescence decay curves of TTV, TTB, TTG, TTY, TTO, TTR, TTDR and TTNIR in the solid state.

The UV-vis absorption spectra of TTV, TTB, TTG, TTY, TTO, TTR, TTDR and TTNIR were measured in acetonitrile (ACN). As shown in Fig. 3A and Table S1,† the solution of building block TTV displays a maximum absorption band at 348 nm, and the maximum absorption peaks of these modified compounds are located ranging from 383 nm to 512 nm. The gradually red-shifted absorption wavelengths can be attributed to the orderly enhanced D–A effect from TTV to TTNIR. To investigate their AIE features, an ACN/H_2_O mixture with different H_2_O fractions was utilized as the solvent system. It was observed that compounds TTB, TTG, TTY, TTO, TTR, TTDR and TTNIR exhibit typical AIE features (Fig. 3C). Taking TTY as an example, there is almost no fluorescence emission when the H_2_O fraction is below 60%. Afterwards, the PL intensity increases dramatically along with raising the fraction of water because of activation of RIM by molecular aggregation and reaches its maximum at 90% water fraction, which is 185-fold higher than that in ACN solution (Fig. 3B). Although the fluorescence intensity of TTV is inversely proportional to the water fraction, its quantum yield in the solid state (27.5%) is higher than that in the solution state (18.6%), definitely demonstrating an aggregation-induced emission enhancement (AIEE) attribute. The gradually decreased fluorescence intensity of TTV along with the increased water fraction could be attributed to its twisted intramolecular charge transfer (TICT) feature,^15^ which was determined by both the red-shifted emission wavelength and the declined emission efficiency accompanying the raised solvent polarity (Fig. S3†). As one of the nonradiative pathways for the excited state to relax and deactivate, the TICT effect is competitive with AIE properties in determining the PL intensity and efficiency using the ACN/H_2_O solution system. In the case of TTV, the AIE feature is strongly depressed by the TICT effect in the nanoaggregation state. As illustrated in Fig. 3D and E and Table S1,† these TPA–thiophene building block-based AIEgens emit efficiently in both nanoaggregation and solid states exhibiting relatively high quantum yields ranging from 3.11% to 40.79%. Each maximum emission wavelength accurately peaks in violet (402 nm), blue (482 nm), green (531 nm), yellow (580 nm), orange (612 nm), red (649 nm), deep red (667 nm) and NIR (724 nm) regions, respectively, suggesting the extremely wide emission color tunability, which is ascribed to both of their varied π-conjugation and D–A effect. Additionally, the fluorescence decay curves in the solid state show that their lifetimes range from 0.64 to 3.69 ns (Fig. 3F and Table S1†).

### Theoretical calculations

**Fig. 4:**
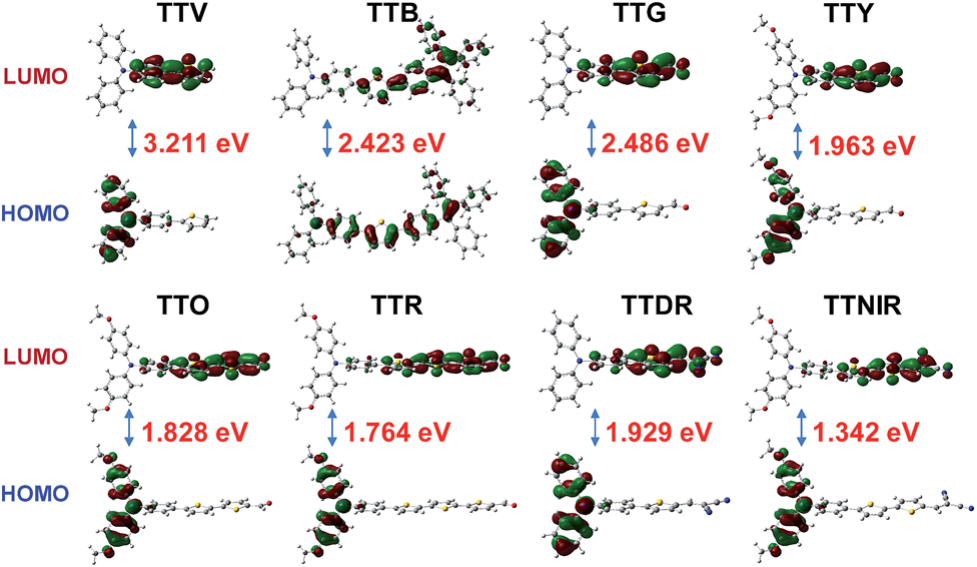
Molecular orbital amplitude plots of HOMO and LUMO energy levels of TTV, TTB, TTG, TTY, TTO, TTR, TTDR and TTNIR calculated at the B3LYP/6-31+G(d) level based on the geometries optimized at the TD-B3LYP/6-31+G(d) level.

To better understand the optical properties of these AIEgens, density functional theory (DFT) calculations were carried out at the B3LYP/6-31+G(d) level with molecular geometries optimized at the TD-B3LYP/6-31+G(d) level (Fig. 4). It was observed that, from TTV to TTNIR, the calculated HOMO–LUMO energy gaps generally decrease, and the results are in good accordance with the experimental data of emission maximums. The orderly declined energy gaps are realized through ingenious modification of the TPA–thiophene building block with diverse electron-donating (thienyl or methoxyl groups) and electron-accepting (aldehyde or cyano groups) units or the π-bridge. Except for TTB, the HOMOs of the remaining AIEgens are delocalized at the TPA moiety, whereas their LUMOs are distributed on the other side of the structures, demonstrating typical D–A structural features. It has been demonstrated that the separation of HOMO and LUMO distributions is essential to effectively reduce the singlet–triplet energy gap, which facilitates the generation of reactive oxygen species (ROS),^16^ further endowing these AIEgens with prominent potential for photodynamic therapy (PDT) applications.^17^ In contrast, TTB possesses an evenly distributed HOMO and LUMO, resulting from its both imperceptible D–A effect and long π-conjugation bridges.

### Bio-imaging, visualization of cell fusion and photodynamic therapy

**Fig. 5:**
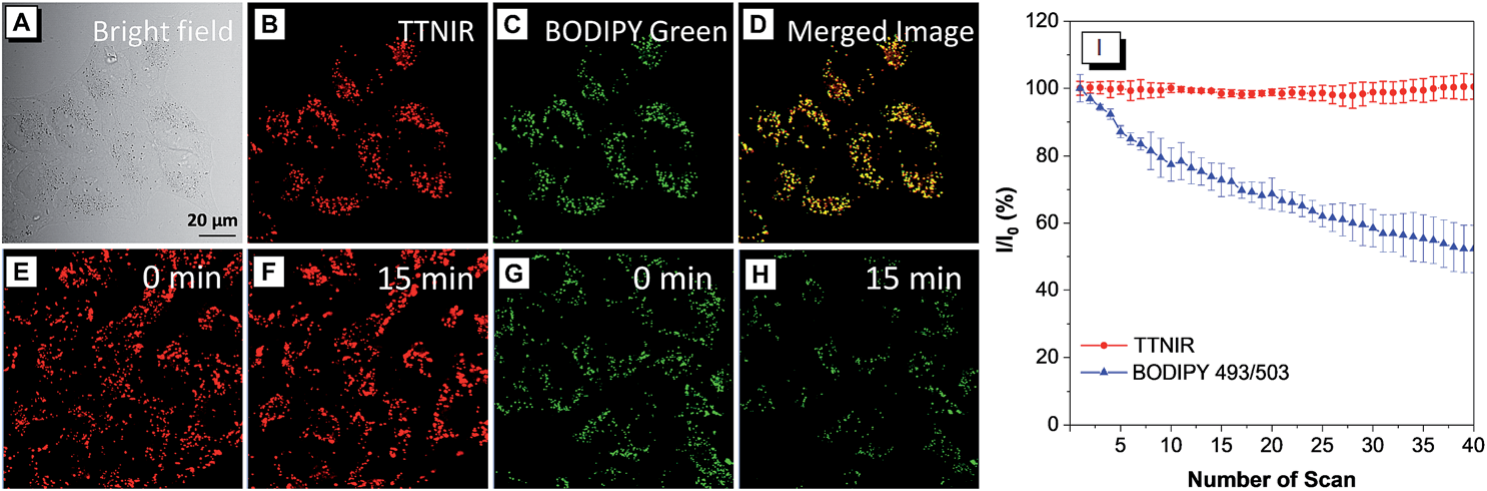
Co-localization imaging of HeLa cells stained with BODIPY493/503 Green and TTNIR, and the investigation of photostability. (A) Bright-field and (B) confocal images of HeLa cells stained with TTNIR and (C) BODIPY493/503 Green. (D) Merged images of panels (B) and (C). *λ*_ex_: 488 nm (1% laser power). Concentrations: TTNIR (1 μM), BODIPY493/503 Green (500 nM). Confocal images of HeLa cells (E and G) before (0 min) and (F and H) after laser irradiation for 15 min and stained with (E and F) TTNIR, and (G and H) BODIPY493/503 Green. (I) Loss in fluorescence of HeLa cells stained with TTNIR and BODIPY493/503 Green with the number of scans of laser irradiation. Scanning rate: 22.4 s per frame. Scale bar = 20 μm.

**Fig. 6:**
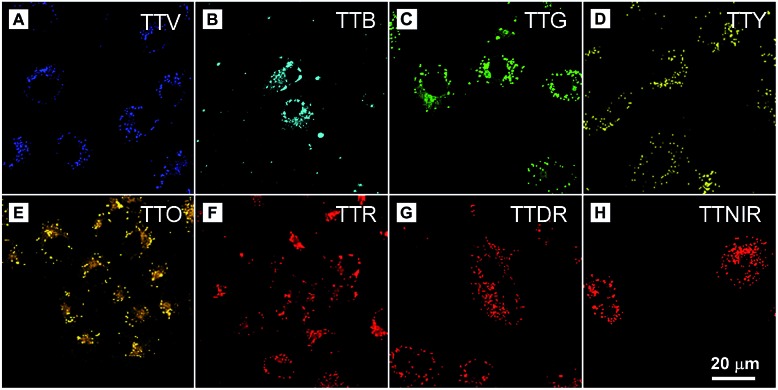
Confocal images of HeLa cells stained with (A) TTV, (B) TTB, (C) TTG, (D) TTY, (E) TTO, (F) TTR, (G) TTDR and (H) TTNIR. Concentration: AIEgen (1 μM).

In the preliminary bioimaging experiment, the cell imaging study was conducted by using HeLa cells as a cell model. Cells were incubated with 1 μM of TTNIR for 20 min; as illustrated in Fig. 5B, bright fluorescence within cells can be observed showing excellent image contrast to the cell background. The co-localization study further proceeded upon incubating HeLa cells with TTNIR and BODIPY493/503 Green. The latter dye is a commercially available bioprobe for the LDs, which are ubiquitous lipid-rich spherical organelles and actively involved in various biofunctions, such as signal transduction, lipid metabolism, and protein degradation. The perfect overlap between TTNIR and BODIPY493/503 Green in cell imaging output indicates the excellent LD-specific targeting capability of TTNIR (Fig. 5B–D). Photostability is a key criterion for evaluating the overall stability of photosensitive substances. The continuous scanning method was then utilized to quantitatively study and compare the photostability of TTNIR and BODIPY493/503 Green. As shown in Fig. 5E–I, after 15 minutes of laser irradiation, the fluorescence intensity of BODIPY493/503 Green encounters an obvious decline, whereas TTNIR shows negligible photobleaching. Moreover, the photostability assessment was also conducted towards Nile Red, which is another commercially available dye for LD-staining (Fig. S13†). It was observed that Nile Red suffered an obvious signal loss with remaining signal intensity around 60% after 15 minutes of laser irradiation, strongly suggesting that the photostability of TTNIR is superior to that of commercially available bioprobes. To further prove its applicability, this staining and imaging strategy using TTNIR is exploited for other cell lines, including NCM460, DLD1, SW480, SW620 and COS-7 (Fig. S4†). In each case after incubation with TTNIR for 20 min, it shows strong and specific internalization into the LDs. Moreover, other AIEgens including TTV, TTB, TTG, TTY, TTO, TTR and TTDR were also investigated for cell imaging. It was observed that LDs can be clearly visualized with excellent image contrast to the cell background through respective incubation of cells with these presented AIEgens (Fig. 6). Pearson's correlation coefficients between AIEgens and commercially available LD-bioprobes were determined to be 90–95%, solidly demonstrating the high specificity of these AIEgens for staining LDs (Fig. S5–S12†). Their excellent LD-staining specificity reasonably results from the lipophilic properties, which bring about efficient accumulation of them in the hydrophobic spherical LDs due to the “like–like” interactions. Evidently, these AIEgens possess various impressive features, such as high brightness, excellent targeting specificities to LDs, extraordinary photostabilities and widely tunable emission colors, making them remarkably important in visualization of biological structures and processes.

**Fig. 7:**
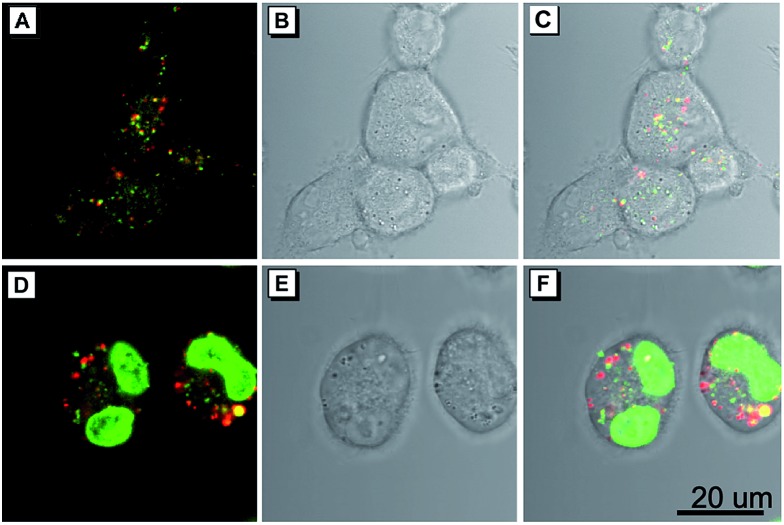
Cell fusion of COS-7 cells induced by 50% polyethylene glycol (PEG), and visualized through fluorescence imaging of TTG and TTNIR. (A) Confocal image and (B) bright-field of mixed cells respectively stained with TTG and TTNIR. (C) Merged images of panels (A) and (B). (D) Confocal image and (E) bright-field of mixed cells respectively stained with TTG, TTNIR and Hoechst 33258. (F) Merged images of panels (D) and (E). For TTG, *λ*_ex_: 405 nm (1% laser power), *λ*_em_: 425–540 nm. For TTNIR, *λ*_ex_: 560 nm (6.5% laser power), *λ*_em_: 600–740 nm. For Hoechst 33258, *λ*_ex_: 405 nm (3.5% laser power), *λ*_em_: 425–540 nm. Concentrations: TTG (500 nM), TTNIR (2 μM), Hoechst 33258 (2.5 μM). Scale bar = 20 μm.

As a common phenomenon in nature, cell fusion is highly associated with many cellular processes, including fertilization, development of placental, regeneration of skeletal muscle, oncogenesis, aneuploidy, chromosomal instability and DNA damage.^18^,^19^ In addition, a recent study shows that cell fusion could play a vital role in alternative therapies for restoring organ function through repairing cellular dysfunction.^19^ Therefore, the development of effective methods for visualizing cell fusion is of great importance. Encouraged by the excellent cell imaging results and homology of the presented AIEgens, a straightforward method for visualization of the cell fusion outcome was conducted by using the combination of TTG and TTNIR as cell imaging agents, due to their minimal overlap of the emission range. In this experiment, two sets of cells were respectively stained with TTG and TTNIR, which were then mingled and treated with polyethylene glycol (PEG) to induce cell fusion.^20^ As illustrated in Fig. 7, after treatment with PEG, both green and red fluorescence of lipid droplets were observed within one single cell, suggesting that cell fusion between TTG- and TTNIR-staining cells successfully proceeded. In addition, the cell fusion outcome was also solidly verified through a commercially available nuclei-staining agent Hoechst 33258. The appearance of two stained nuclei within one single cell (Fig. 7D) indicated that the visualization strategy of the cell fusion outcome by using two AIEgens with different emission ranges is definitely reliable. Evidently, the developed AIEgens having widely tunable emissions and high emission efficiencies are potentially useful in the fundamental study of cell fusion.

**Fig. 8:**
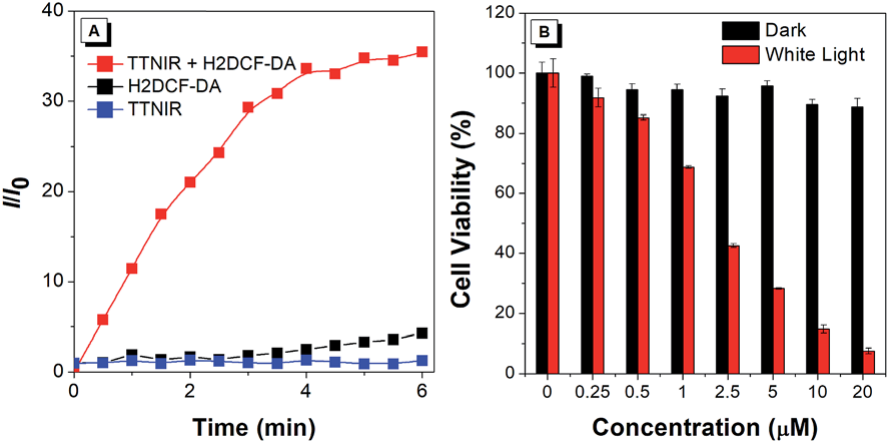
ROS generation upon white light irradiation and PDT study of TTNIR. (A) Relative change in fluorescence intensity (*I*/*I*_0_) at 534 nm of H2DCF-DA, TTNIR, and mixtures of TTNIR and H2DCF-DA in PBS upon white light irradiation for different times. Concentrations: 10 μM (TTNIR) and 5 μM (H2DCF-DA). (B) Cell viability of HeLa cells stained with different concentrations of TTNIR in the absence or presence of white light irradiation.

Intense fluorescence in the near-infrared (NIR) region is highly desirable for many clinical processes, due to the salient advantages of deep tissue penetration, minimal photodamage to biological structures, and high image contrast to the physiological background.^21^ Moreover, NIR emission is generally realized by intensifying the D–A effect of the structure, resulting in the separation of HOMO and LUMO distribution, as well as the decrease of the singlet–triplet energy gap, thus facilitating the generation efficiency of ROS. Therefore, the AIEgen TTNIR with both bright NIR emission and the strong D–A effect is potentially efficient for PDT, which is an extraordinary therapeutic modality, and has captivated much interest for treating various malignant and non-malignant diseases with minimal invasiveness and precise controllability. In the preliminary test, the ROS generation efficiency of TTNIR was investigated using H2DCF-DA as the indicator, which can emit fluorescence at around 534 nm triggered by ROS. As shown in Fig. 8A, in the presence of TTNIR, the emission of H2DCF-DA was rapidly intensified with the increase of irradiation time using white light as the irradiation source, reaching 36-fold enhancement in 6 min compared with the original emission intensity. In contrast, the fluorescence intensities of AIEgens or H2DCF-DA alone were very low and remained almost constant under the same irradiation conditions. These results reveal good photo-sensitizing properties for ROS generation. Quantitative evaluation of the phototherapy effect of TTNIR on HeLa cells was then explored through the standard 3-(4,5-dimethylthiazol-2-yl)-2,5-diphenyltetrazolium bromide (MTT) assay. The dose-dependent toxicity study shows that there is no obvious cytotoxicity observed for the HeLa cells treated with TTNIR in the dark, even with the TTNIR concentration reaching as high as 20 μM (Fig. 8B). Upon white light exposure, cell viability dropped gradually with raising the concentration of TTNIR. Only 7% of cell viability remained with utilizing 20 μM of TTNIR, demonstrating almost complete cell apoptosis. Apparently, TTNIR holds high effectiveness for cancer cell ablation by means of PDT.

## Conclusions

To sum up, we report the first series of AIEgens with widely tunable emissions covering the whole visible region extending to the NIR area. These TPA–thiophene building block-based AIEgens can be facilely prepared by extremely simple synthetic protocols, and show high fluorescence quantum yields up to 40.79% in the solid state benefiting from their intrinsic aggregation-induced emission nature. They have been successfully utilized for LD-specific cell imaging, showing excellent image contrast to the cell background and higher photostability than the commercial LD-staining fluorophore. Additionally, the high brightness and homology of these AIEgens endow them with excellent performance for visualizing cell fusion. To the best of our knowledge, this would be the first report on using AIEgens as fluorescent probes for assessing cell fusion. Notably, upon exposure to white light irradiation, one of these presented AIEgens, namely TTNIR, displays high ROS generation efficiency, enabling its effective application for photodynamic ablation of cancer cells.

Our findings in this study provide an ideal fluorescence system for widely tuning emission colors with high brightness at will. This successful example would further facilitate the exploration of organic fluorophores with AIE features for preclinical research and clinical applications.

## Experimental procedures

### Materials and methods

Chemicals for synthesis were purchased from Sigma-Aldrich, MERYER or J&K, and used without further purification. All solvents were purified and dried following standard procedures. ^1^H spectra were measured on Bruker ARX 400 NMR spectrometers using CD_2_Cl_2_ or CDCl_3_ as the deuterated solvent. Mass spectrometric measurements (HRMS) were performed on a Finnigan MAT TSQ 7000 mass spectrometer system operating in matrix-assisted laser desorption/ionization time of flight mass spectrometry (MALDI-TOF) mode. UV-vis spectra were measured on a Milton Ray Spectronic 3000 array spectrophotometer. Steady-state photoluminescence (PL) spectra were recorded on a PerkinElmer LS 55 spectrophotometer. Fluorescence images of AIEgens in the solid state and aggregation state were collected on an Olympus BX 41 fluorescence microscope. The cellular fluorescence images were taken using a Zeiss laser scanning confocal microscope (LSM7 DUO) and analyzed using ZEN 2009 software (Carl Zeiss).

### Synthesis of compound TTV7c,^22^

A mixture of the bromide substituted triphenylamine moiety (1.2 mmol), thiophen-2-ylboronic acid moiety (1 mmol), THF (20 mL), K_2_CO_3_ aqueous solution (2 M, 1.6 mL), and Pd(PPh_3_)_4_ (58 mg, 0.05 mmol) was degassed and charged with N_2_. The mixture was refluxed overnight. The reaction was quenched by the addition of water (30 mL) and extracted with CH_2_Cl_2_ (3 × 30 mL). The combined organic layer was dried over anhydrous Na_2_SO_4_ and evaporated. The residue was purified by column chromatography over silica gel using petroleum ether to afford the desired product TTV with a yield of 78%. ^1^H NMR (400 MHz, CD_2_Cl_2_): 7.60 (d, *J* = 6.8 Hz, 2H), 7.41 (d, *J* = 8 Hz, 2H), 7.37–7.33 (m, 4H), 7.13–7.06 (m, 9H). ^13^C NMR (100 MHz, CDCl_3_): 147.49, 147.20, 144.26, 129.27, 128.54, 127.95, 126.71, 124.42, 123.98, 123.75, 123.02, 122.21. ESI HRMS: calcd. for C_22_H_17_NS [M]^+^: 327.1082, found: 327.1066.

### Cell imaging and confocal co-localization

In 35 mm glass-bottomed dishes, the cells (NCM460, DLD1, SW480, and SW620) were seeded and cultured at 37 °C. After incubation with TTNIR (1 μM) for 20 min, the cells were washed with PBS three times and subjected to imaging analysis using a laser scanning confocal microscope (Zeiss Laser Scanning Confocal Microscope; LSM7 DUO). The excitation filter was 488 nm and the emission filter was 570–740 nm. For the co-staining assay, the AIEgen loaded COS-7 cells were subjected to incubation with BODIPY 493/503 Green or Nile red for 20 min. Afterwards, the cells were washed with PBS and then observed with CLSM. The cells were imaged using appropriate excitation and emission filters for each dye. The co-localization efficiency was analyzed with Olympus FV10-ASW software, in which the calculated Pearson's coefficient was above 0.90.

### Photostability

For the photostability test, the cells were imaged using a confocal microscope (Zeiss Laser Scanning Confocal Microscope; LSM7 DUO) and analyzed using ZEN 2009 software (Carl Zeiss). Both TTNIR and BODIPY493/503 Green were excited at 488 nm for one-photon imaging (1% laser power). The scanning speed was 22.4 s per scan, and the repeated image scans were taken 40 times. The first scan of both TTNIR and BODIPY493/503 Green was set to 100%, followed by which the pixel intensity values were averaged and plotted against the scan number. The resulting curve represents the bleaching rate.

### ROS generation and PDT study

H2DCF-DA was used as the ROS generation indicator. In the experiments, 10 μL of H2DCF-DA stock solution (1.0 mM) was added to 2 mL of TTNIR suspension, and white light (18 mW cm^–2^) was employed as the irradiation source. The emission of H2DCF-DA at 534 nm was recorded at various irradiation periods. HeLa cells were seeded in 96-well plates (Costar, IL, USA) at a density of 6000–8000 cells per well. After overnight cell culture, the medium in each well was replaced with 100 mL fresh medium containing different concentrations of TTNIR. Following 30 min incubation, the plates containing HeLa cells were exposed to white light (around 18 mW cm^–2^) for 30 min, and another array of plates with cells were kept in the dark as the control.

### Cell fusion

Two dishes of COS-7 cells were incubated with TTG and TTNIR for half an hour separately. After that the cells were washed with PBS 3 times, collected by adding trypsin, and centrifuged respectively. Then the cells were mixed together and incubated for 2 hours in another Petri dish with a cover glass. 10 g of polyethylene glycol 3400 was dissolved in 10 mL of Dulbecco's modified Eagle's medium (DMEM) without FBS. The mixed culture was overlaid for 5 min at 37 °C with 2 mL PEG solution. Then the PEG solution was gradually diluted with DMEM in four steps at the interval of 2 min, by adding 0.5, 1, 2, and 4 mL DMEM, respectively, after which the liquid was removed and replaced with DMEM.

## Conflicts of interest

There are no conflicts to declare.

## Supplementary Material

Supplementary information

Crystal structure data
