# Supplementary material for: Facile synthesis of AIEgens with wide color tunability for cellular imaging and therapy
Source: Chem Sci. 2019 Feb 22;10(12):3494–501. doi: 10.1039/c8sc05805a (PMC6432335; doi:10.1039/c8sc05805a)
Supplement: Supplementary file 1 [file SC-010-C8SC05805A-s001.pdf]

Electronic Supplementary Material (ESI) for Chemical Science.
This journal is © The Royal Society of Chemistry 2019

Supporting Information

Facile synthesis of AIEgens with wide color tunability for cellular imaging and therapy

Wenhan Xu, Michelle M. S. Lee, Zhihan Zhang, Herman H. Y. Sung, Ian D. Williams, Ryan T. K. Kwok, Jacky W. Y. Lam, Dong Wang, Ben Zhong Tang**

Table of Contents

Experimental procedures
NMR and HRMS spectra
Table S1. Optical properties of AIEgens
Figure S1**.** Single crystal structure of TTY
Figure S2. Single crystal structure of TTDR
Table S2-S8. Crystallographic data collection and structure refinement details of TTG
Table S9-S16. Crystallographic data collection and structure refinement details of TTY
Figure S3. Solvatochromism effect evaluation of TTV and TTG
Figure S4. Extension of the LDs-specific imaging strategy using TTNIR to stain different cell types.
Figure S5. Colocalization imaging of COS-7 cells stained with TTV and Nile Red
Figure S6. Colocalization imaging of COS-7 cells stained with TTB and Nile Red.
Figure S7. Colocalization imaging of COS-7 cells stained with TTG and Nile Red.
Figure S8. Colocalization imaging of COS-7 cells stained with TTY and Nile Red.
Figure S9. Colocalization imaging of COS-7 cells stained with TTO and BODIPY493/503 Green.
Figure S10. Colocalization imaging of COS-7 cells stained with TTR and BODIPY493/503 Green.
Figure S11. Colocalization imaging of COS-7 cells stained with TTDR and BODIPY493/503 Green.
Figure S12. Colocalization imaging of COS-7 cells stained with TTNIR and BODIPY493/503 Green.
Figure S13. Photostability investigation of TTNIR compared with BODIPY493/503 Green and Nile Red.
Figure S14. MTT study of cell viability treated with 50% PEG.

Experimental procedures

Synthesis of compound TTG

The synthetic process was similar to TTV except for the change of starting materials. ^{1}H NMR (400 MHz, $CDCl_3$): 9.85 (s, 1H), 7.70 (d, *J* = 4 Hz, 1H), 7.52 (d, *J* = 8.8 Hz, 2H), 7.31-7.28 (m, 5H), 7.14-7.05 (m, 8H). ^{13}C NMR (100 MHz, $CDCl_3$): 182.57, 154.55, 149.11, 146.94, 141.28, 137.70, 129.46, 127.22, 126.10, 125.14, 123.85, 122.83, 122.33. ESI HRMS: calcd. for $C_{23}H_{17}NOS$ $[M]^+$: 355.1031, found: 355.1030.

Synthesis of compound TTY

The synthetic process was similar to TTV except for the change of starting materials. ^{1}H NMR (400 MHz, $CDCl_3$): 9.83 (s, 1H), 7.69 (d, *J* = 4 Hz, 1H), 7.46 (d, *J* = 9.2 Hz, 2H), 7.25 (d, *J* = 4.8 Hz, 1H), 7.10-7.08 (m, 4H), 6.91-6.85 (m, 6H), 3.81 (s, 6H). ^{13}C NMR (100 MHz, $CDCl_3$): 182.47, 156.51, 155.06, 149.98, 140.78, 139.85, 137.79, 127.19, 127.11, 124.21, 122.27, 119.23, 114.85, 55.46. ESI HRMS: calcd. for $C_{25}H_{21}NO_3S$ $[M]^+$: 415.1242, found: 415.1248.

Synthesis of compound TTO

The synthetic process was similar to TTV except for the change of starting materials. ^{1}H NMR (400 MHz, $CDCl_3$): 9.84 (s, 1H), 7.65 (d, *J* = 4 Hz, 1H), 7.39 (d, *J* = 9.2 Hz, 2H), 7.30 (d, *J* = 4 Hz, 1H), 7.21 (d, *J* = 4 Hz, 1H), 7.13-7.07 (m, 5H), 6.92-6.84 (m, 6H), 3.81 (s, 6H). ^{13}C NMR (100 MHz, $CDCl_3$): 182.33, 156.24, 148.95, 147.60, 146.72, 141.072, 137.44, 133.48, 127.22, 126.90, 126.46, 125.13, 123.54, 122.60, 119.94, 114.79, 55.47. ESI HRMS: calcd. for $C_{29}H_{23}NO_3S_2$ $[M]^+$: 497.1119, found: 497.1127.

Synthesis of compound TTR

The synthetic process was similar to TTV except for the change of starting materials. ^{1}H NMR (400 MHz, $CDCl_3$): 9.86 (s, 1H), 7.67 (d, *J* = 4Hz, 1H), 7.40 (d, *J* = 8.8 Hz, 2H), 7.27 (d, *J* = 3.2 Hz, 1H), 7.23 (d, *J* = 4 Hz, 1H), 7.16 (d, *J* = 4 Hz, 1H), 7.11-6.08 (m, 6H), 6.92 (d, *J* = 8.8 Hz,

2H), 6.86 (d, J = 8.8 Hz, 4H), 3.82 (s, 6H). ^{13}C NMR (100 MHz, $CDCl_3$): 182.34, 156.13, 148.61, 146.95, 144.74, 141.42, 140.41, 139.56, 137.37, 134.05, 133.93, 126.97, 126.79, 126.31, 125.57, 125.42, 124.05, 123.87, 122.39, 120.17, 114.76, 55.47. ESI HRMS: calcd. for $C_{31}H_{21}NOS_3$ $[M]^+$: 519.0785, found: 579.0761.

Synthesis of compound 4-(5-bromothiophen-2-yl)-N,N-diphenylaniline

4-Borate triphenylamine (742 mg, 2.0 mmol), 2,5-dibromothiophene (423 mg, 1.8 mmol), K_2CO_3 aqueous solution (2 M, 2.4 mL), $Pd(PPh_3)_4$ (116 mg, 0.1 mmol) was placed in a 100 mL two-neck round bottom flask and 30 mL of THF was added as solvent under nitrogen. The mixture was heated to reflux for 10 h, cooled to room temperature, transferred to 40 mL of saturated salt water, extracted with DCM (40 mL × 3), filtered under reduced pressure, and the crude product was purified by column chromatography (petroleum ether/ethyl acetate = 40/1) to give pale yellow solid (583 mg, 72% yield). ^1H NMR (400 MHz, $CDCl_3$) δ 7.39 (d, J = 8.7 Hz, 2H), 7.33–7.26 (m, 4H), 7.16-7.12 (m, 4H), 7.11-7.05 (m, 4H), 7.02 (d, J = 3.8 Hz, 1H), 6.97 (d, J = 3.8 Hz, 1H).

Synthesis of compound TTB

Under nitrogen, 4-(5-bromothiophen-2-yl)-*N*,*N*-diphenylaniline (406 mg, 1 mmol), TPE-$B(OH)_2$ (451 mg, 1.2 mmol), $Pd(PPh_3)_4$ (58 mg, 0.05 mmol), K_2CO_3 aqueous solution (2 M, 0.8 mL) in 20 ml THF were heated to reflux overnight. After cooling to room temperature, the product was extracted with dichloromethane. After removal of the solvent, the crude product was purified on a silica gel column using hexane/ethyl acetate = 20/1 as eluent to give yellow solid. ^1H NMR (400 MHz, $CDCl_3$): 7.47 (d, J = 8.4 Hz, 2H), 7.37 (d, J = 8.4 Hz, 2H), 7.30-7.27 (m, 4H), 7.22 (d, J = 4.0 Hz, 1H), 7.17 (d, J = 4.0 Hz, 1H), 7.15-7.0 (m, 25H). ^{13}C NMR (100 MHz, $CDCl_3$): 147.65, 147.44, 143.91, 143.86, 143.79, 143.47, 143.06, 142.69, 141.36, 140.58, 132.47, 132.08, 131.61, 131.54, 129.51, 128.55, 128.03, 127.91, 127.83, 126.76, 126.72, 126.64, 126.54, 124.77,

124.70, 124.04, 123.85, 123.30. ESI HRMS: calcd. for $C_{48}H_{35}NS$ $[M]^+$: 657.2490, found: 657.2491.

Synthesis of compound TTDR

A mixture of TTG (1.0 mmol), malononitrile (1.1 mmol) in ethanol (3 mL) was heated to reflux for 72 h. After cooling down to room temperature, the solvent was removed under vacuum. Then water (20 mL) was added into the mixture, which was extracted with CH_2Cl_2 (1 mL × 3). The combined organic phase was dried over Na_2SO_4 and filtered; the filtrate was removed under reduced pressure in order to obtain the crude product, which was further purified by silica gel chromatography (petroleum ether/CH_2Cl_2 as eluent) to give product with the yield of 49%. ^1H NMR (400 MHz, $CDCl_3$): 7.74 (s, 1H), 7.68 (d, J = 4 Hz, 1H), 7.52 (d, J = 8.8 Hz, 2H), 7.33-7.30 (m, 5H), 7.16-7.10 (m, 6H), 7.04 (d, J = 8.8 Hz, 2H). ^{13}C NMR (100 MHz, $CDCl_3$): 157.11, 150.24, 149.91, 146.60, 140.34, 133.06, 129.56, 127.54, 125.48, 124.82, 124.32, 123.25, 121.68, 114.54, 113.66, 75.01. ESI HRMS: calcd. for $C_{26}H_{17}N_3S$ $[M]^+$: 403.1143, found: 403.1131.

Synthesis of compound TTNIR

The synthetic process was similar to TTDR except for the change of starting reactant from TTG to TTO. ^1H NMR (400 MHz, $CDCl_3$): 7.72 (s, 1H), 7.61 (d, J = 4 Hz, 1H), 7.41-7.37 (m, 3H), 7.24 (d, J = 4 Hz, 1H), 7.16 (d, J = 4 Hz, 1H), 7.10-7.07 (m, 4H), 6.91-6.85 (m, 6H), 3.81 (s, 6H). ^{13}C NMR (100 MHz, $CDCl_3$): 156.37, 149.96, 149.88, 149.29, 148.38, 140.28, 140.06, 132.87, 132.48, 128.53, 127.04. 126.54, 124.58, 123.85, 122.92, 119.64, 114.82, 114.45, 113.62, 55.47. ESI HRMS: calcd. for $C_{32}H_{23}N_3O_2S_2$ $[M]^+$: 545.1232, found: 545.1241.

NMR and HRMS spectra

^{1}H NMR spectrum of TTV:

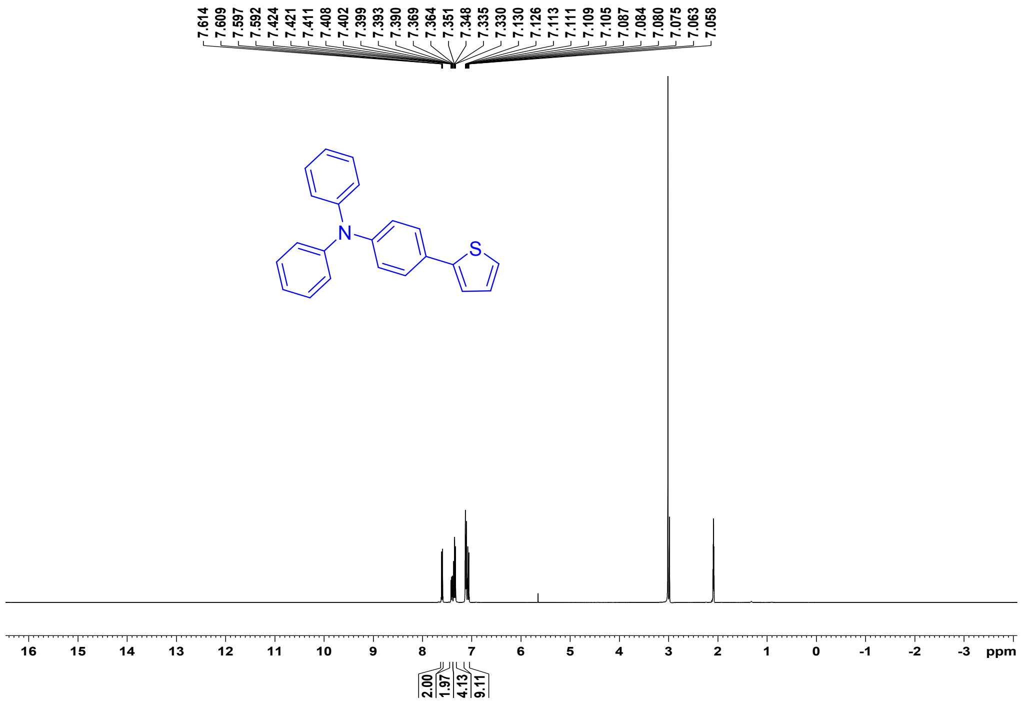

^{13}C NMR spectrum of TTV:

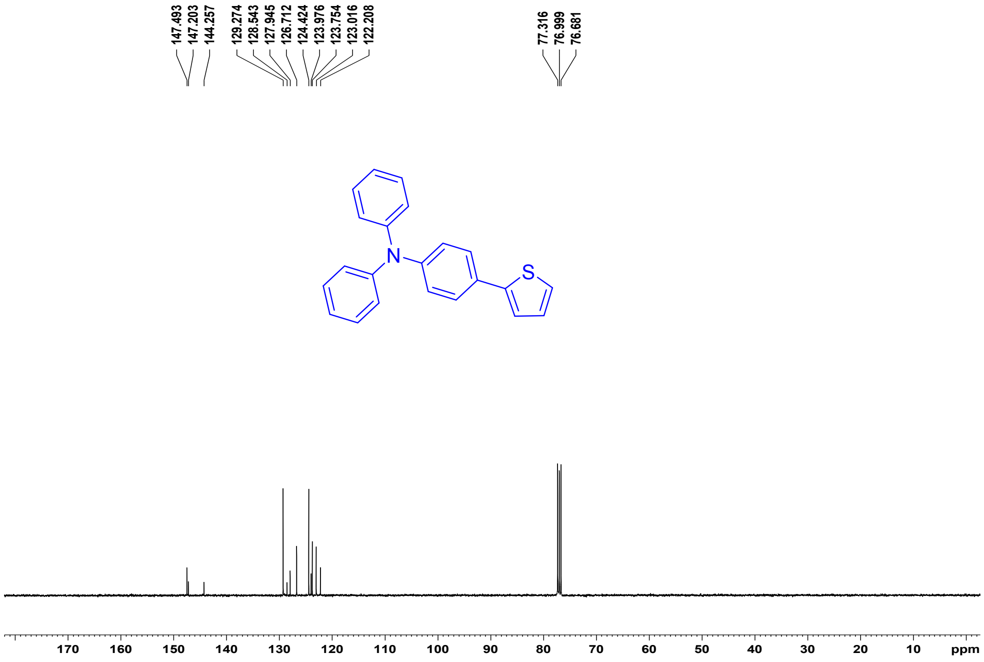

HRMS spectrum of TTV:

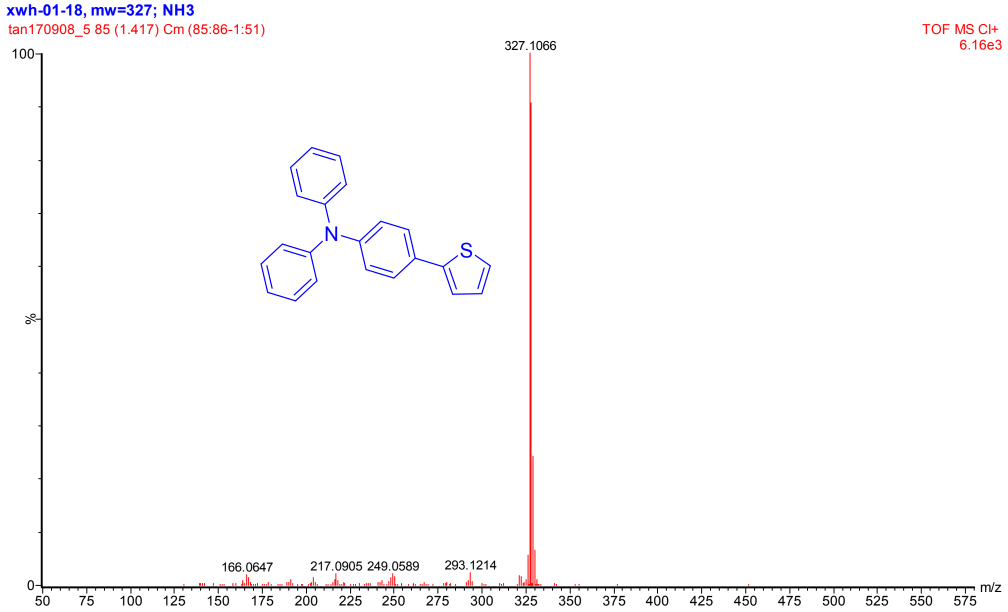

^{1}H NMR spectrum of TTB:

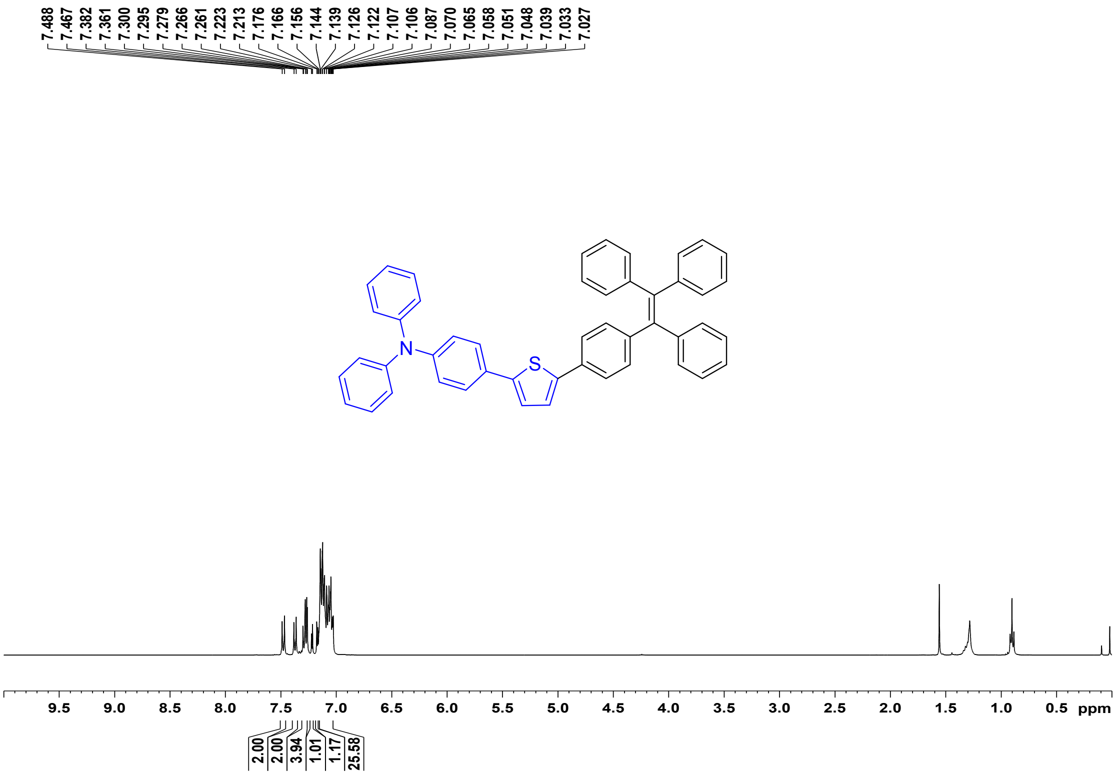

^{13}C NMR spectrum of TTB:

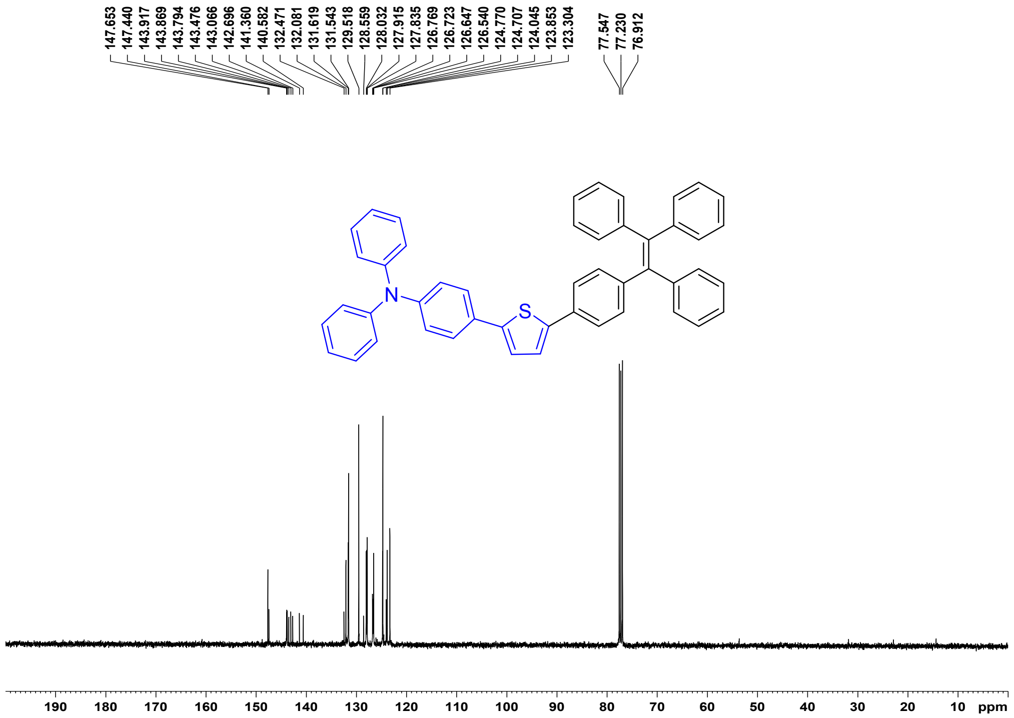

HRMS spectrum of TTB:

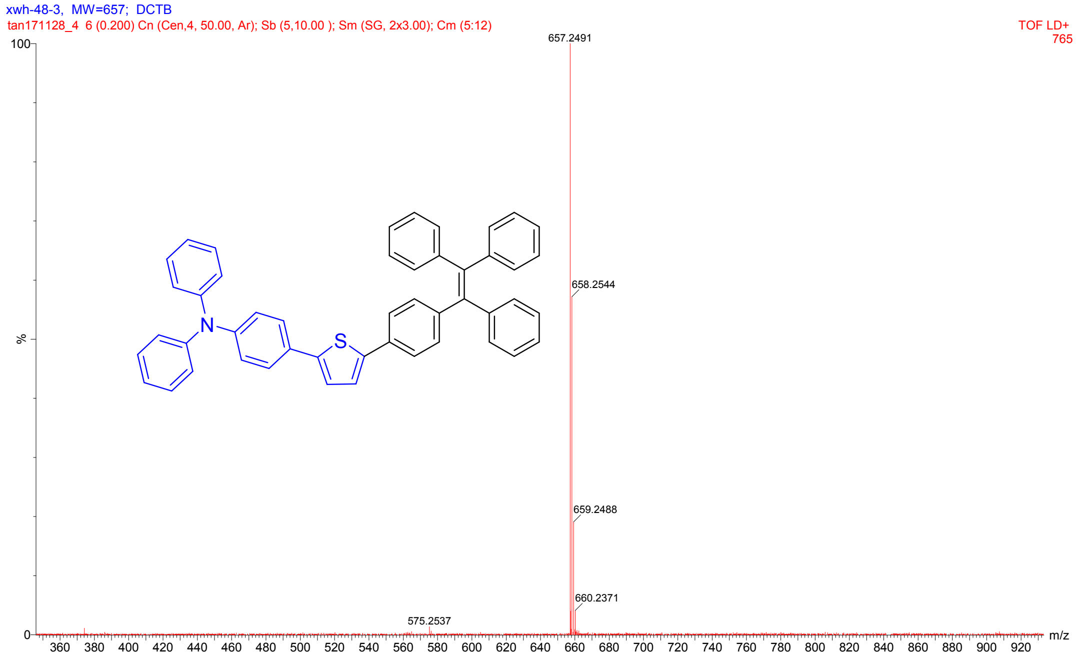

^{1}H NMR spectrum of TTG:

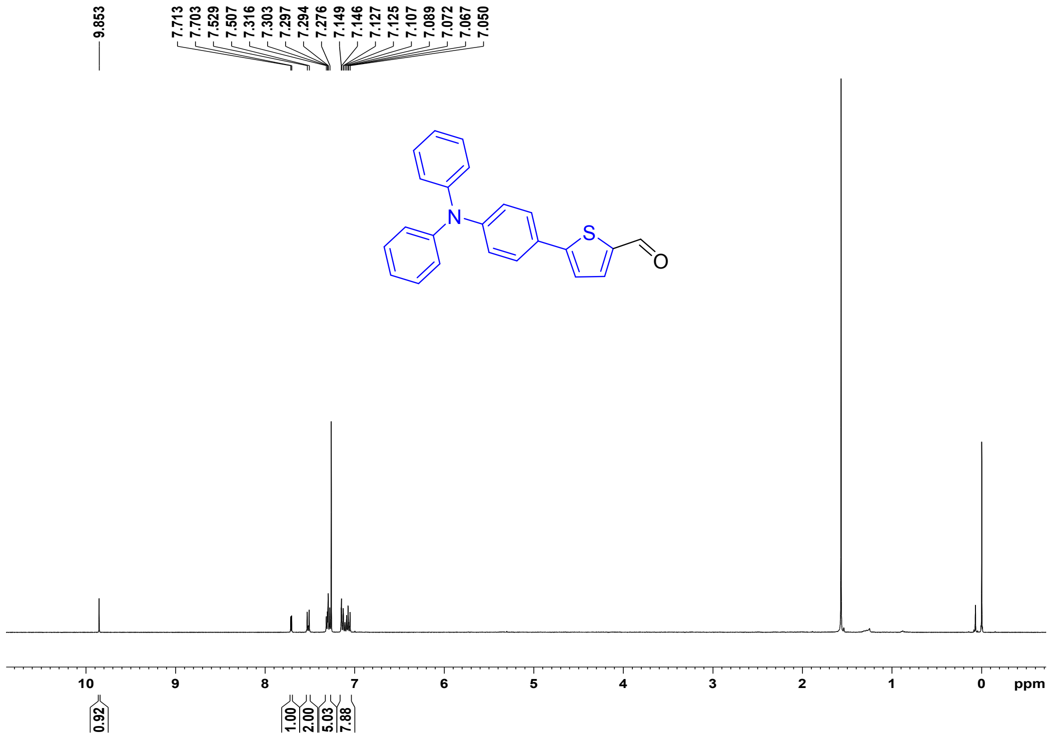

^{13}C NMR spectrum of TTG:

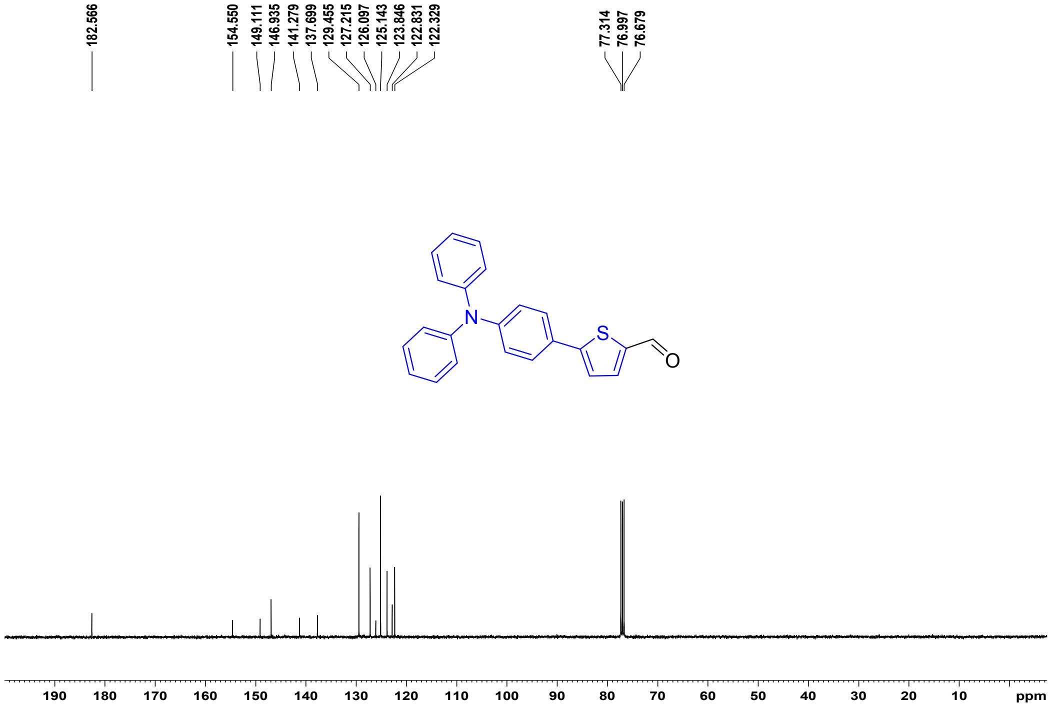

HRMS spectrum of TTG:

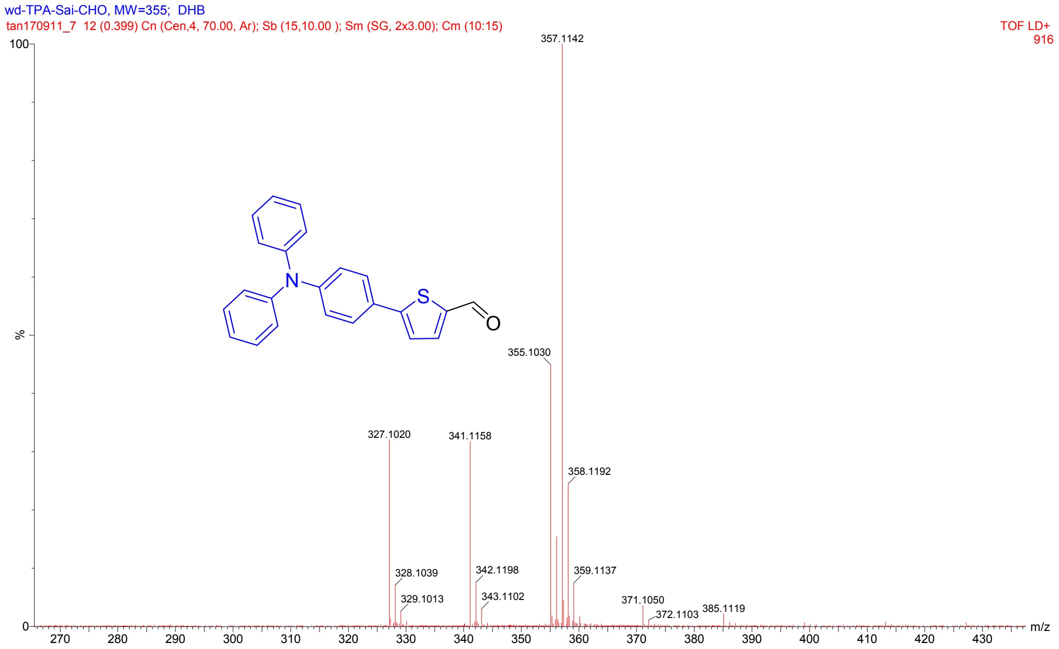

^1H NMR spectrum of TTY:

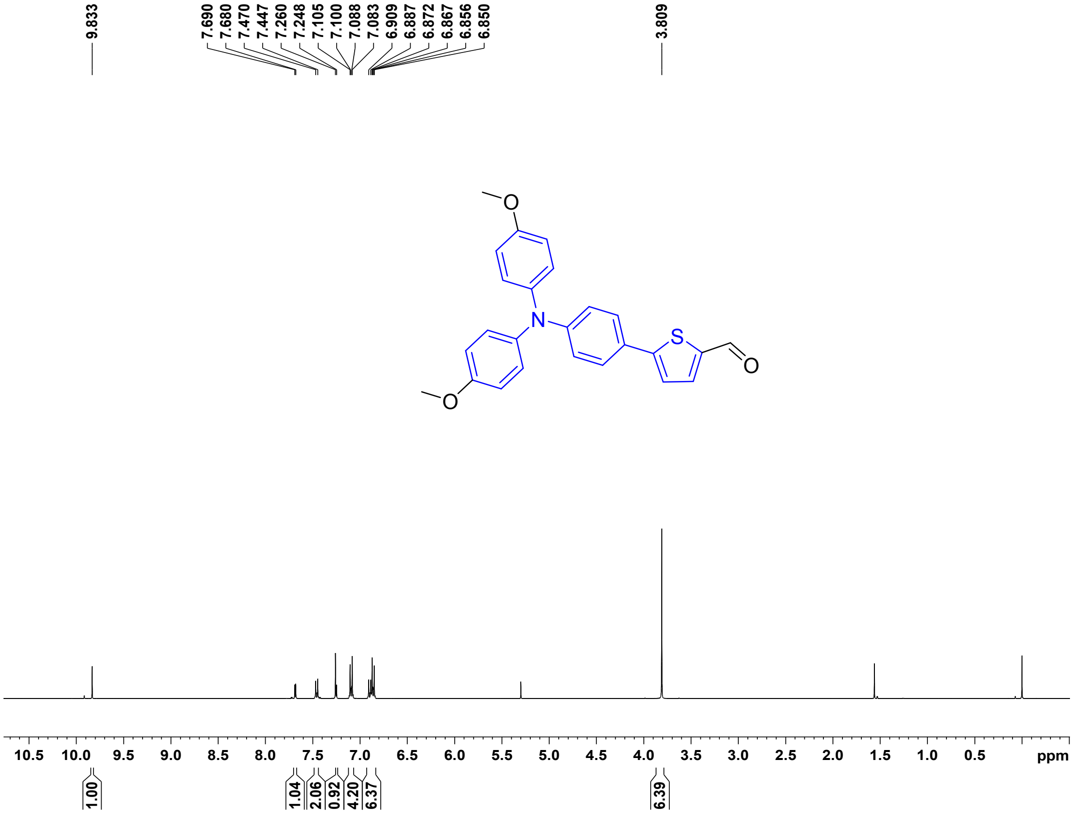

[13] C NMR spectrum of TTY:

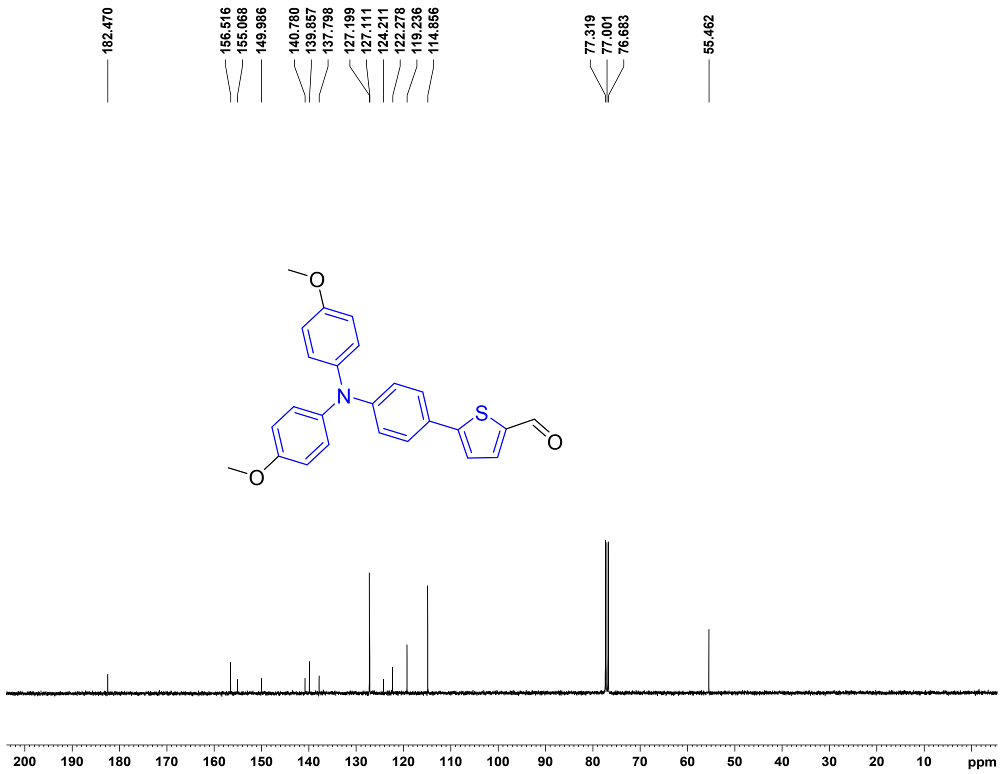

HRMS spectrum of TTY:

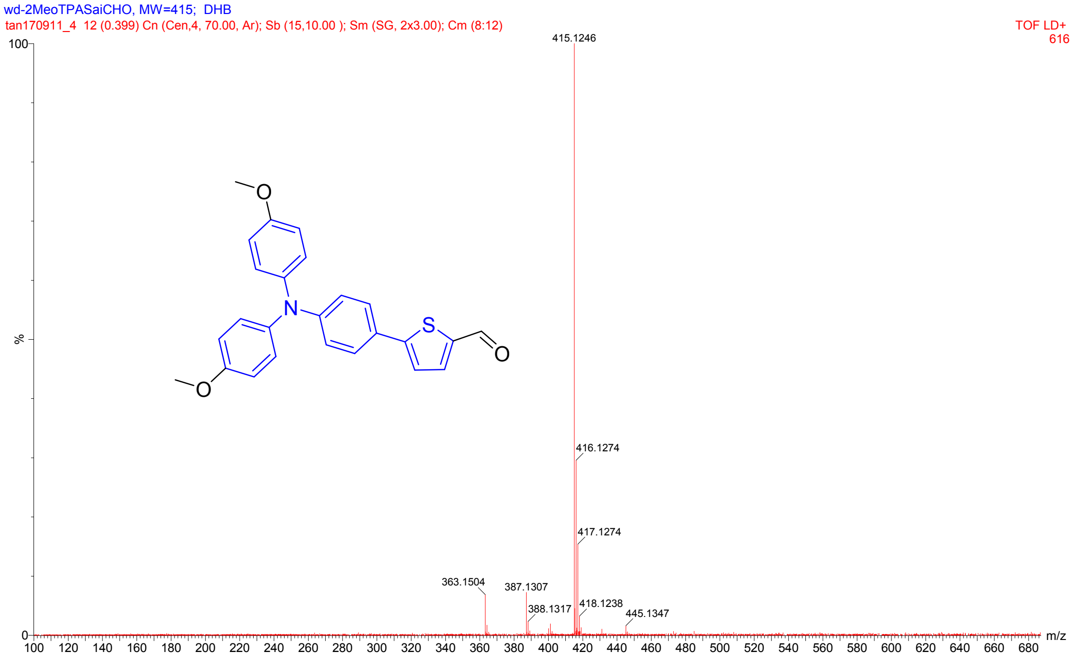

^{1}H NMR spectrum of TTO:

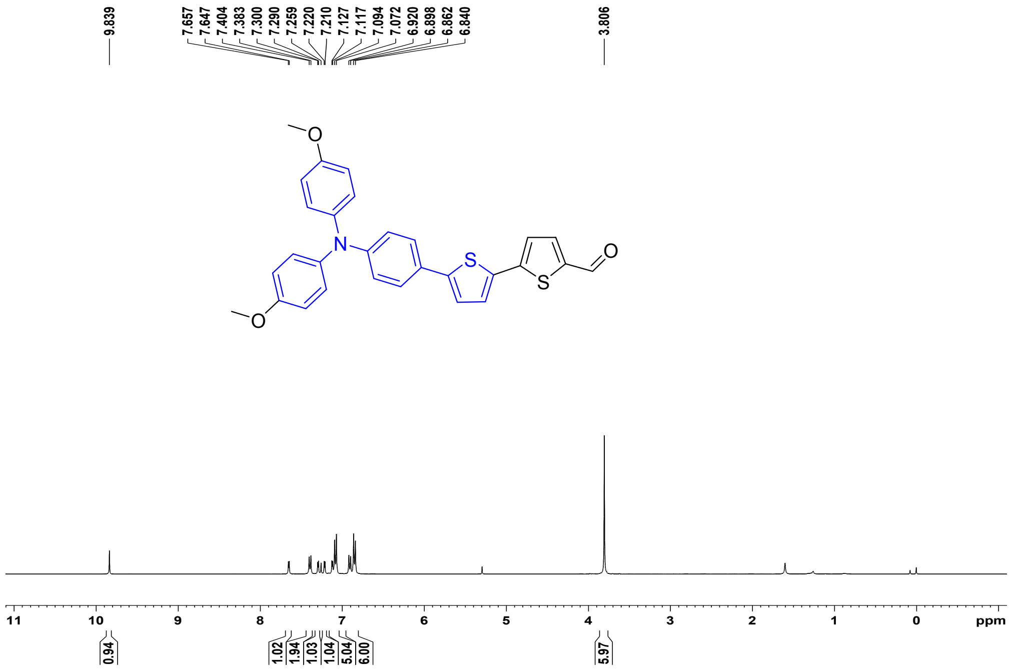

^{13}C NMR spectrum of TTO:

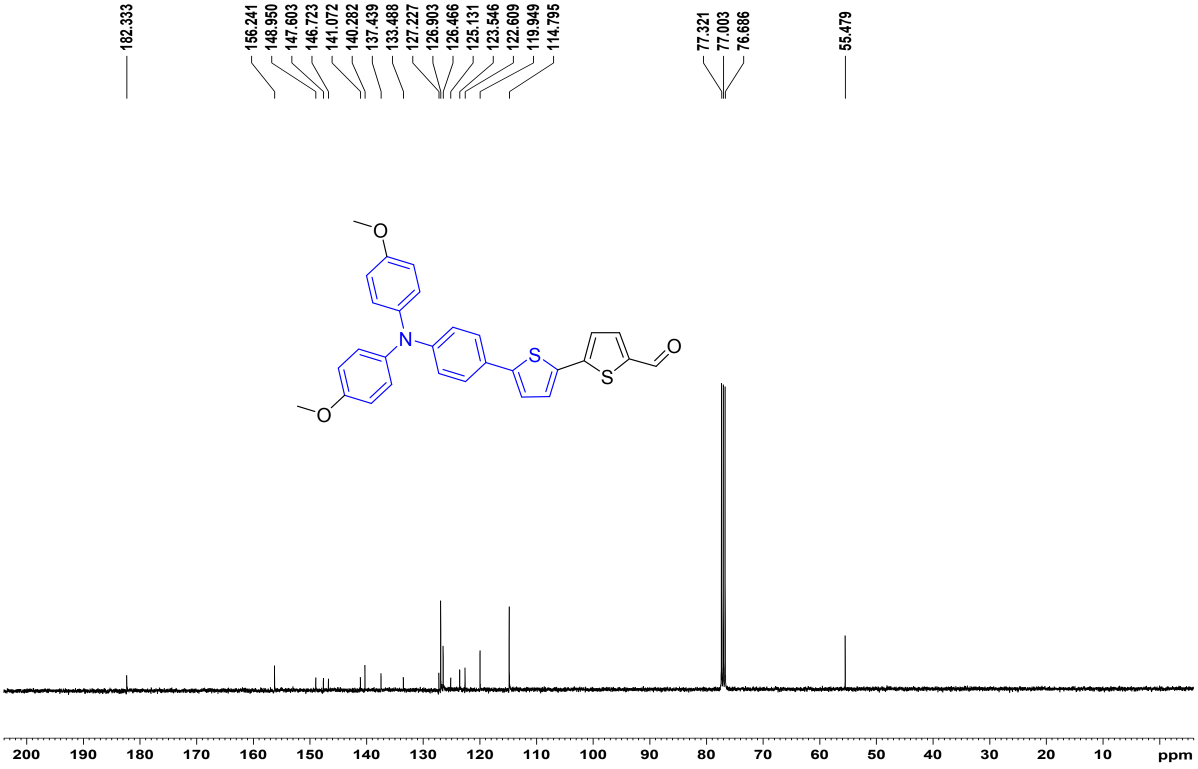

HRMS spectrum of TTO:

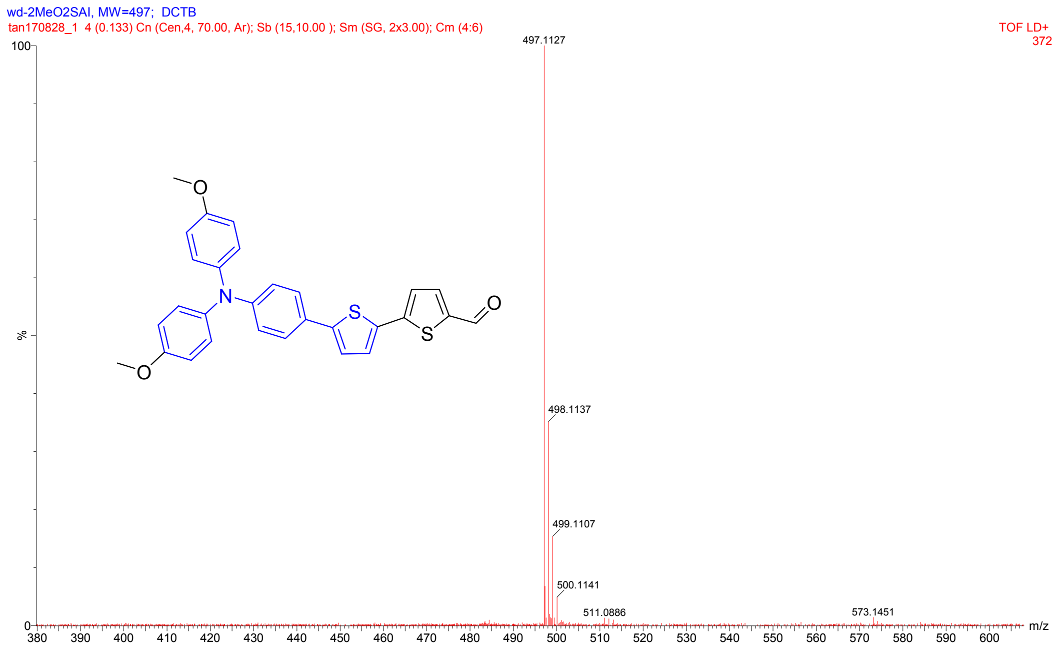

^{1}H NMR spectrum of TTR:

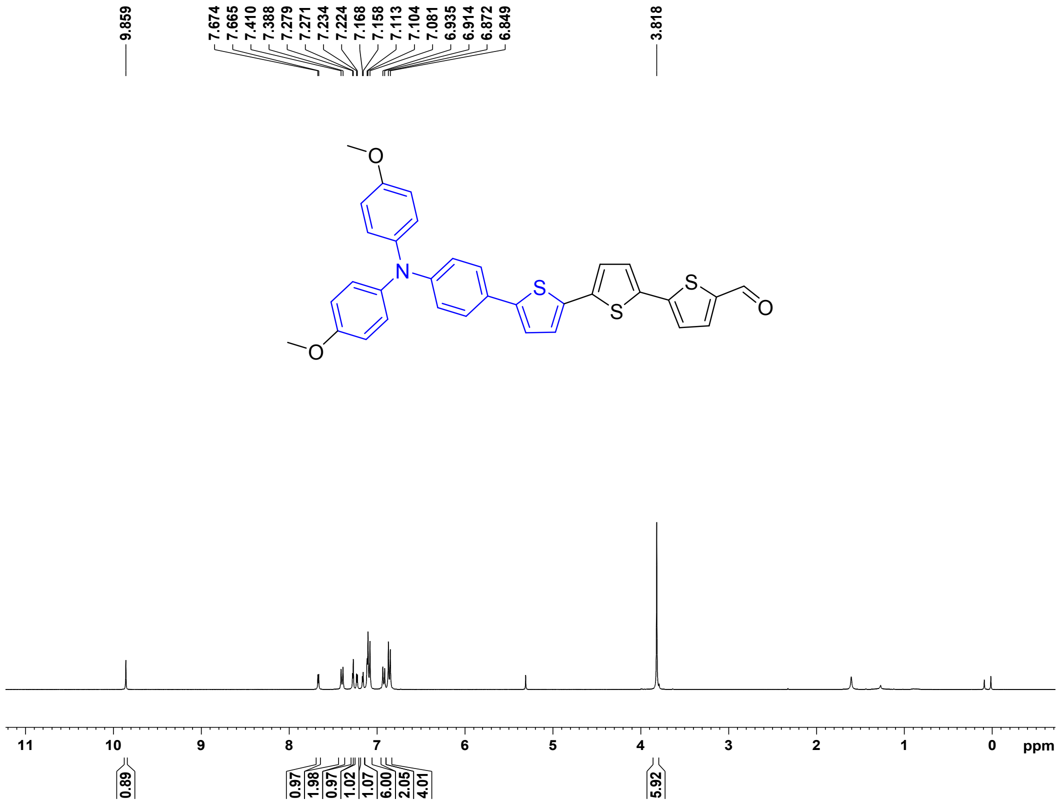

^{13}C NMR spectrum of TTR:

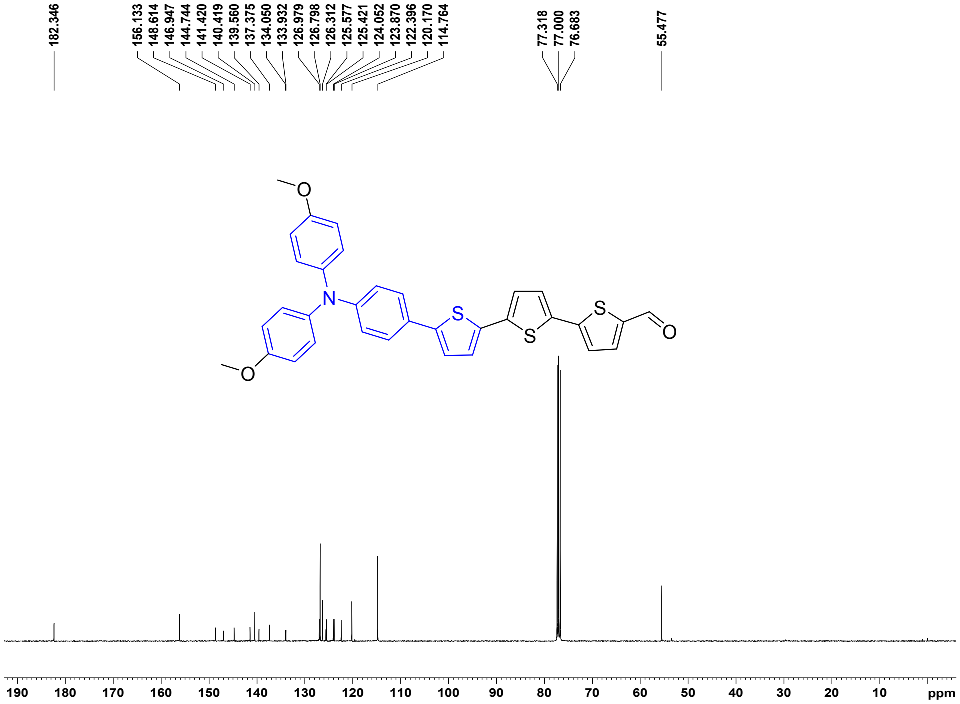

HRMS spectrum of TTR:

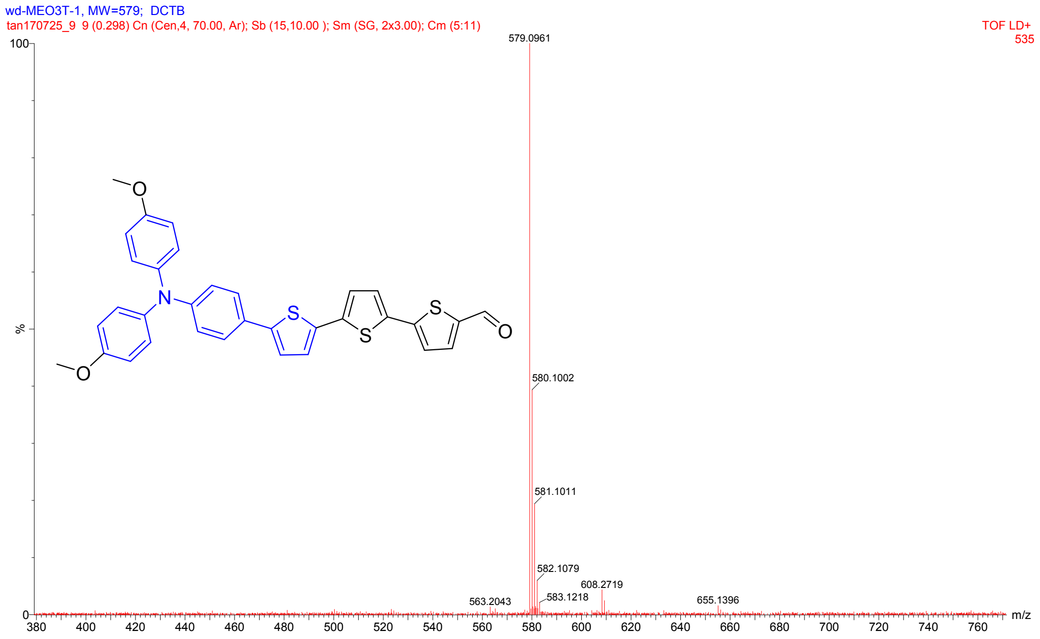

^{1}H NMR spectrum of TTDR:

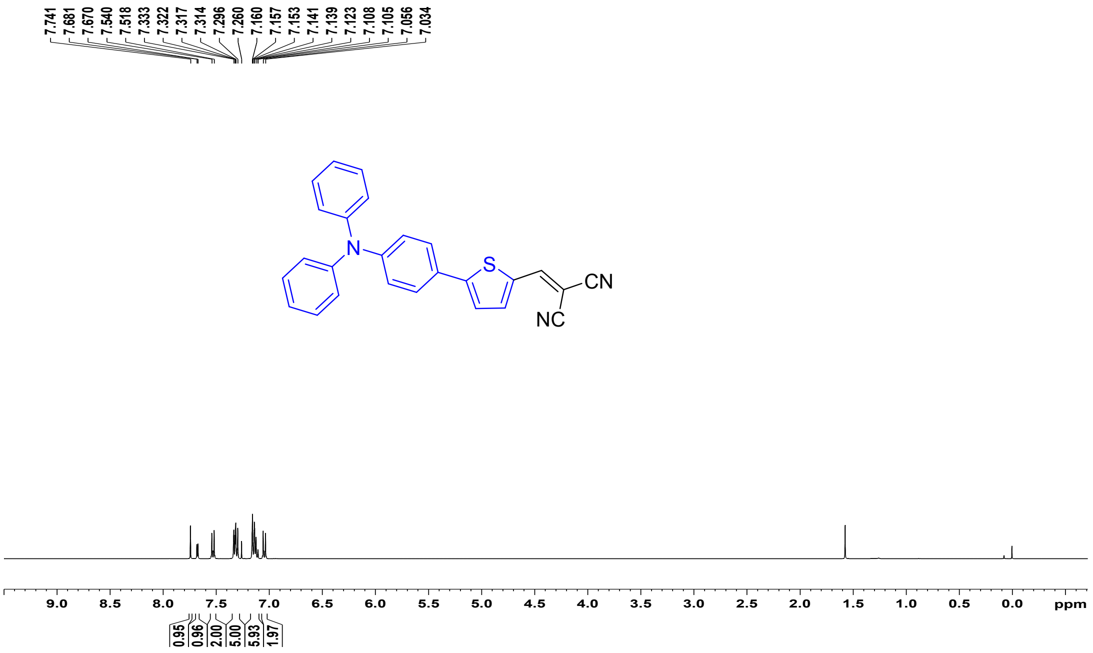

^{13}C NMR spectrum of TTDR:

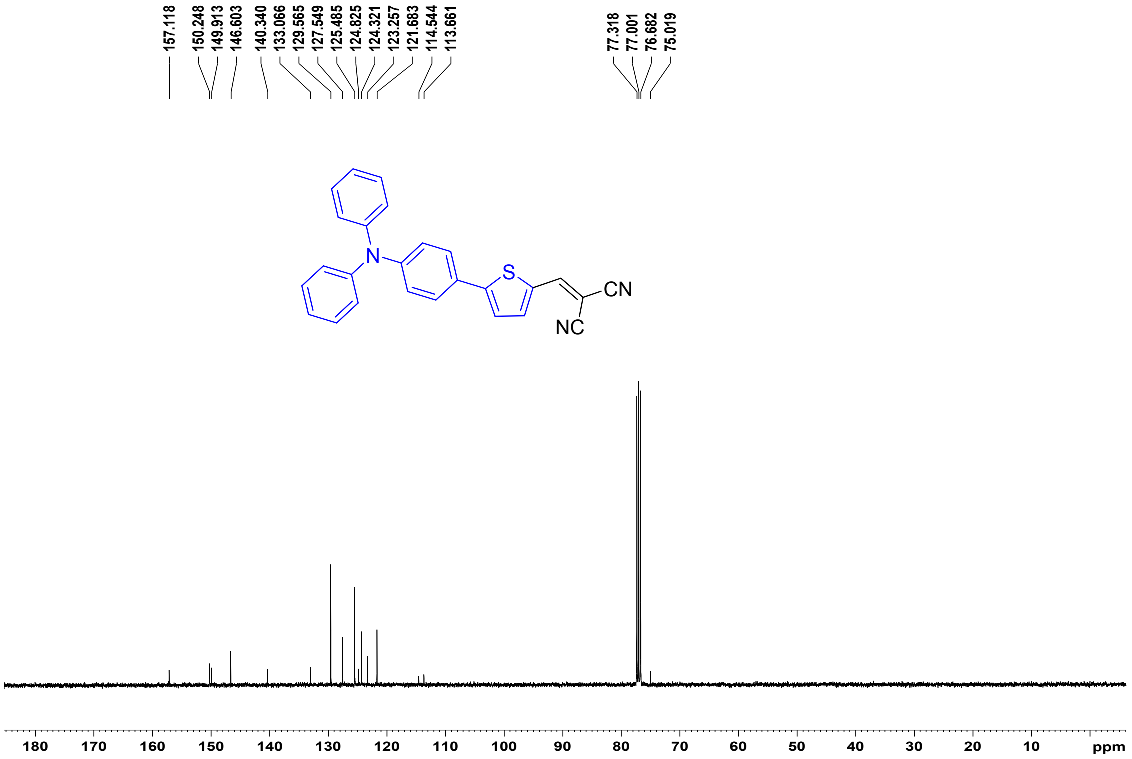

HRMS spectrum of TTDR:

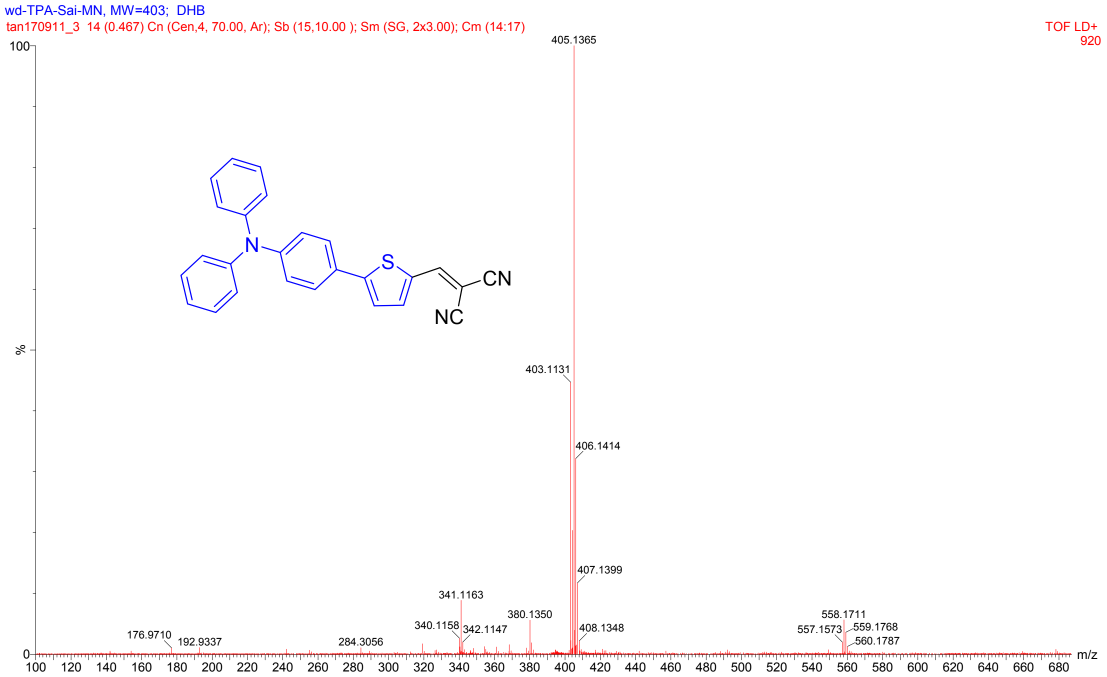

^{1}H NMR spectrum of TTNIR:

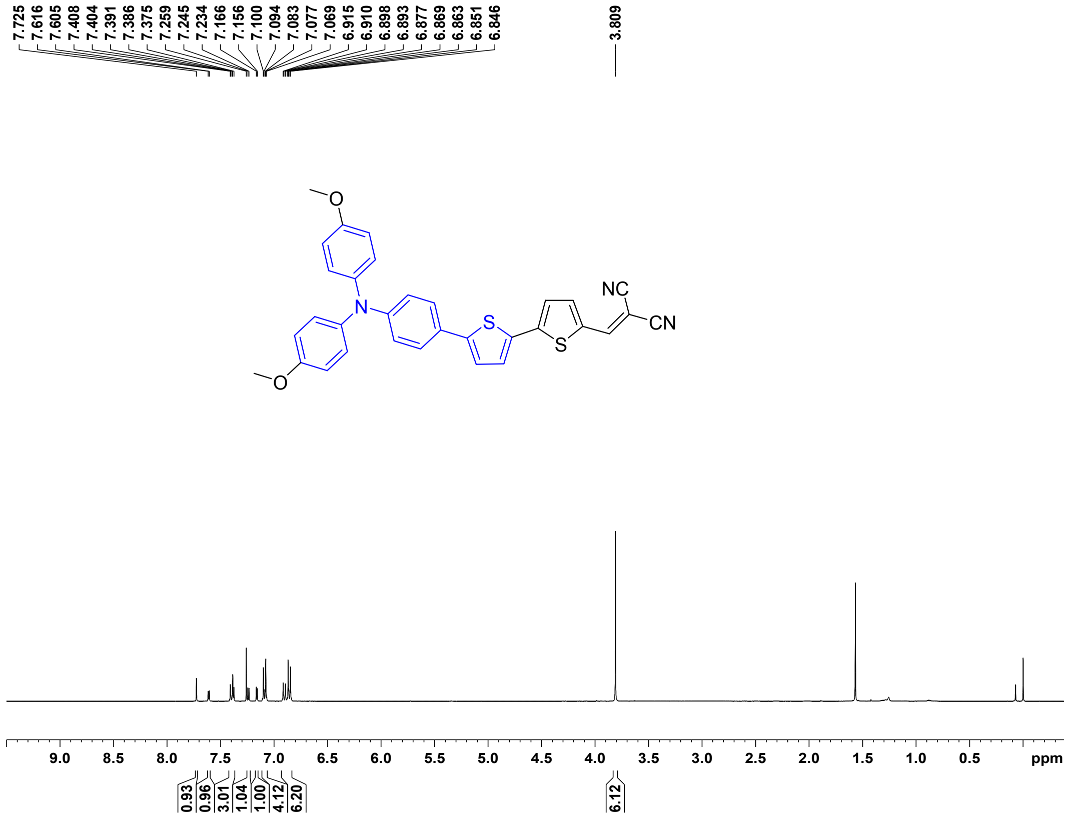

^{13}C NMR spectrum of TTNIR:

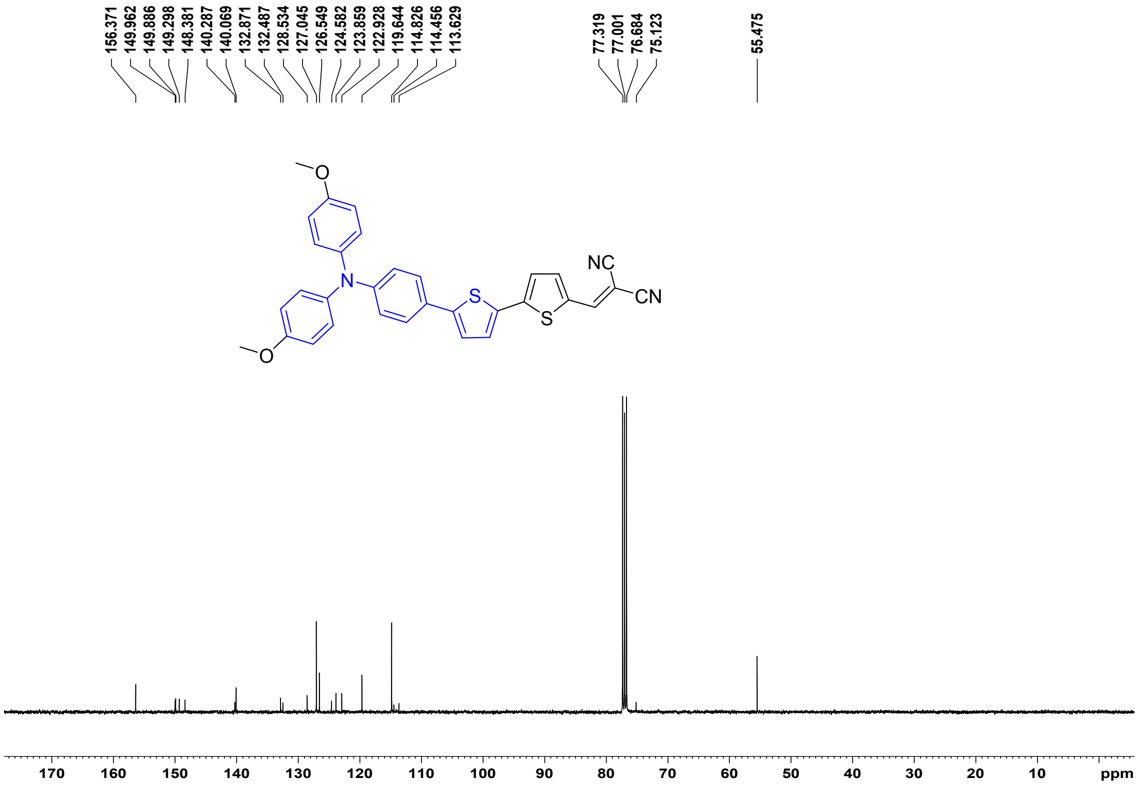

HRMS spectrum of TTNIR:

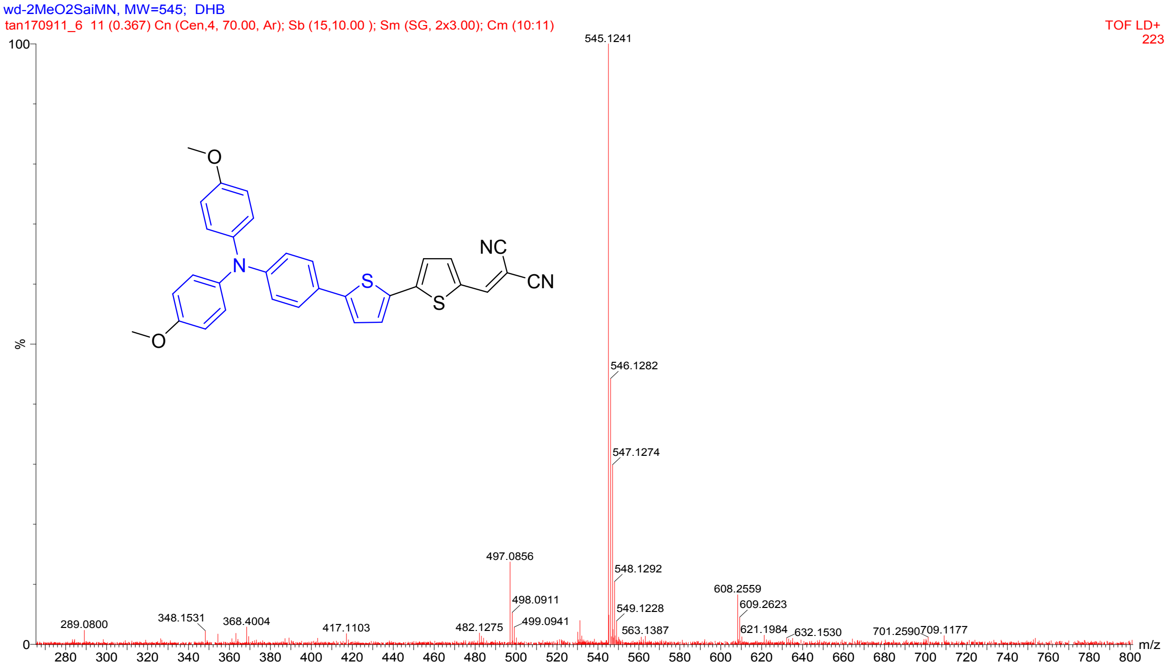

Table S1. Optical properties of AIEgens TTV, TTB, TTG, TTY, TTO, TTR, TTDR and TTNIR.

AIEgens	Solution				Solid			
	λ_{abs} [nm][a)]	ε [×10^4M^{-1}cm^{-1}]	λ_{em} [nm] (Φ_F[%]) (Soln.)[b,e)]	λ_{em} [nm] (Φ_F[%]) (Aggr.)[b,d)]	λ_{em} [nm] (Solid.)[c)]	$I_{aggr,max}/I_{soln}$	Φ_F[%][b)]	τ [ns][f)]
TTV	348	5.7	429 (18.6)	402 (14.1)	417	-	27.54	1.41
TTB	383	4.4	502 (2.9)	482 (34.5)	489	15.7	40.79	1.31
TTG	398	3.7	564 (0.6)	531 (10.2)	539	103	26.04	3.69
TTY	410	3.5	614 (0.1)	580 (8.3)	583	185	10.03	1.22
TTO	438	10.9	538 (1.4)	612 (7.3)	603	8.1	7.57	1.37
TTR	443	3.8	555 (0.3)	649 (1.2)	659	3.9	6.12	0.64
TTDR	484	6.6	664 (0.6)	667 (5.8)	684	11.6	18.3	2.42
TTNIR	512	4.9	626 (0.2)	724 (0.4)	706	2.4	3.11	0.85

[a)] Absorption maximum in ACN solutions; [b)] All the fluorescence quantum yields are determined by a calibrated integrating sphere system; [c)] Emission maximum in solid state; [d)] Quantum yields of aggregation state are collected with 95% water fraction; [e)] Emission maximum in ACN (10 μM); [f)] Fluorescence lifetime, measured under ambient conditions.

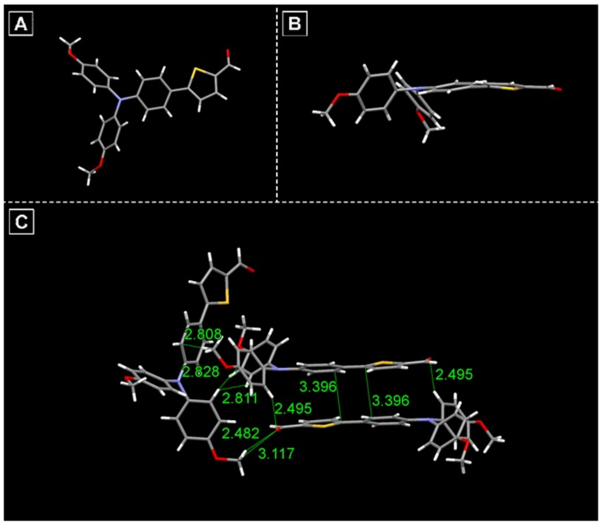

Figure S1. A) Single crystal structure of TTY. B) Side view of the crystal structure of TTY. C) Various inter- and intramolecular interactions in crystals of TTY.

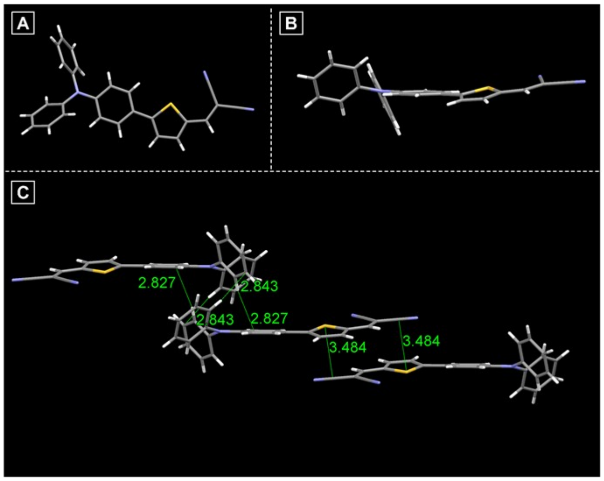

Figure S2. A) Single crystal structure of TTDR. B) Side view of the crystal structure of TTDR. C) Various inter- and intramolecular interactions in crystals of TTDR.

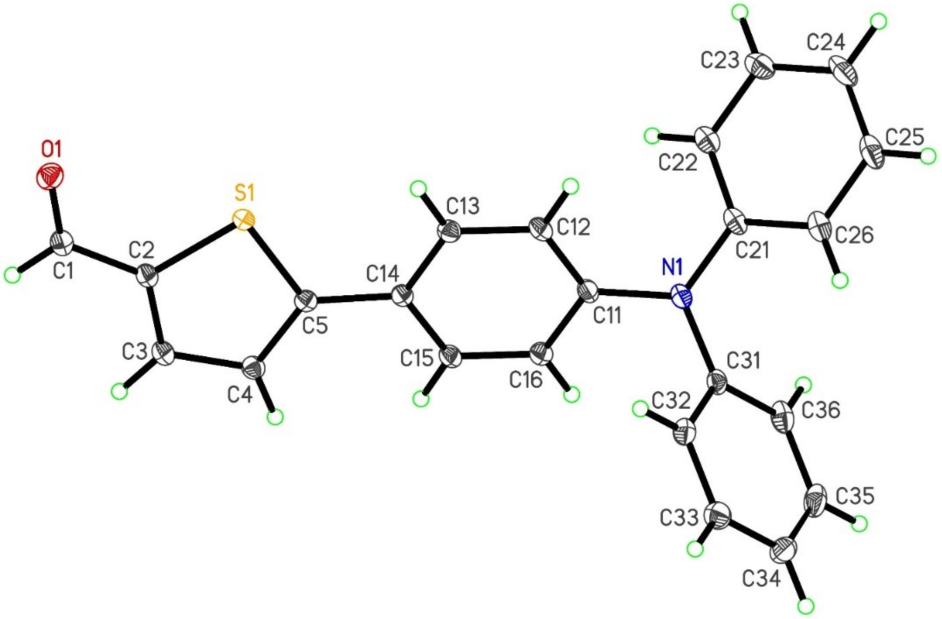

Table S2. Crystal data and structure refinement for TTG.

Identification code	TTG
Empirical formula	$C_{23}H_{17}NOS$
Formula weight	355.43
Temperature/K	100.00(10)
Crystal system	monoclinic
Space group	$P2_1/c$
a/Å	19.92637(17)
b/Å	6.63771(5)

c/Å	13.28322(11)
α/°	90
β/°	96.4721(8)
γ/°	90
Volume/$Å^3$	1745.71(2)
Z	4
ρ_{calc}g/cm^3	1.352
μ/mm^{-1}	1.724
F(000)	744.0
Crystal size/mm^3	0.4 × 0.04 × 0.02
Radiation	CuKα (λ = 1.54184)
2Θ range for data collection/°	8.932 to 134.984
Index ranges	-23 ≤ h ≤ 23, -7 ≤ k ≤ 7, -7 ≤ l ≤ 15
Reflections collected	8579
Independent reflections	3114 [R_{int} = 0.0220, R_{sigma} = 0.0203]
Data/restraints/parameters	3114/0/235
Completeness to theta = 66.5°	99.4%
Goodness-of-fit on F^2	1.004
Final R indexes [I>=2σ (I)]	R_1 = 0.0358, wR_2 = 0.0988
Final R indexes [all data]	R_1 = 0.0363, wR_2 = 0.0995
Largest diff. peak/hole / e Å^{-3}	0.31/-0.43

Table S3. Fractional Atomic Coordinates (×10^4) and Equivalent Isotropic Displacement Parameters (Å^2×10^3) for TTG. U_{eq} is defined as 1/3 of of the trace of the orthogonalised U_{IJ} tensor.

**Atom**	***x***	***y***	***z***	**U(eq)**
S1	4923.4(2)	-405.8(4)	3629.5(2)	15.24(13)
O1	6209.3(5)	-2992.5(14)	3542.1(7)	21.3(2)
N1	1959.9(6)	4237.0(18)	4253.9(9)	19.4(3)
C1	6291.4(7)	-1181(2)	3612.5(9)	17.6(3)
C2	5761.7(7)	289(2)	3683.2(9)	16.0(3)
C3	5839.4(7)	2331(2)	3826.9(10)	17.2(3)
C4	5226.9(6)	3329(2)	3906.5(9)	16.5(3)
C5	4677.5(6)	2046.2(19)	3814.3(9)	14.6(3)
C11	2617.7(6)	3610(2)	4079.3(9)	16.6(3)
C12	2808.0(6)	1594(2)	4036.8(10)	17.7(3)
C13	3471.1(7)	1088(2)	3927.0(9)	16.7(3)
C14	3968.5(6)	2567(2)	3877.0(9)	14.4(3)
C15	3769.4(7)	4582.4(19)	3907.4(10)	16.1(3)
C16	3108.1(7)	5095(2)	4001(1)	17.3(3)
C21	1372.6(7)	3164(2)	3871.6(10)	18.6(3)
C22	1356.0(7)	2048(2)	2979.3(11)	20.5(3)

C23	782.2(8)	966(2)	2614.7(11)	25.3(3)
C24	209.2(7)	1007(2)	3124.1(13)	29.3(3)
C25	221.5(7)	2143(2)	3998.6(13)	28.0(3)
C26	794.2(7)	3213(2)	4378.9(11)	22.6(3)
C31	1929.1(6)	5696(2)	5041.2(10)	17.0(3)
C32	2311.2(7)	5391(2)	5973.6(11)	19.5(3)
C33	2296.5(7)	6800(2)	6740.1(11)	24.5(3)
C34	1900.3(8)	8506(2)	6594.7(12)	27.1(3)
C35	1521.9(7)	8817(2)	5666.2(13)	27.8(3)
C36	1536.5(7)	7429(2)	4884.0(11)	23.0(3)

Table S4. Anisotropic Displacement Parameters (Å^2×10^3) for TTG. The Anisotropic displacement factor exponent takes the form: $-2\pi^2[h^2a^{*2}U_{11}+2hka^*b^*U_{12}+\ldots]$.

Atom	U_{11}	U_{22}	U_{33}	U_{23}	U_{13}	U_{12}
S1	13.5(2)	15.4(2)	16.7(2)	-1.62(10)	1.37(13)	-1.29(10)
O1	23.1(5)	19.4(5)	21.2(5)	-0.3(4)	1.7(4)	2.7(4)
N1	12.2(6)	24.2(6)	21.7(6)	-3.8(5)	1.8(4)	-1.9(5)
C1	16.1(6)	23.7(7)	13.1(6)	1.4(5)	2.0(5)	-0.1(5)
C2	13.6(6)	21.0(7)	13.4(6)	0.8(5)	1.5(5)	-1.6(5)
C3	15.0(6)	20.3(7)	16.4(6)	1.6(5)	1.5(5)	-2.4(5)
C4	16.4(6)	16.5(6)	16.7(6)	0.8(5)	2.1(5)	-1.4(5)
C5	17.0(7)	17.3(6)	9.4(6)	0.7(4)	0.9(5)	-0.7(5)
C11	13.4(6)	22.4(7)	13.9(6)	-1.4(5)	1.0(5)	-1.5(5)
C12	15.1(6)	19.3(7)	18.6(6)	0.4(5)	1.4(5)	-4.2(5)
C13	17.1(6)	16.3(6)	16.5(6)	0.0(5)	0.9(5)	-1.0(5)
C14	14.8(6)	18.7(6)	9.8(6)	-0.1(5)	1.3(5)	-0.7(5)
C15	14.6(6)	17.9(7)	15.9(6)	0.2(5)	2.0(5)	-3.5(5)
C16	16.9(7)	16.5(6)	18.4(7)	-0.5(5)	1.8(5)	-0.7(5)
C21	13.7(6)	19.3(7)	22.0(7)	5.6(5)	-1.5(5)	-1.5(5)
C22	16.8(6)	21.1(7)	22.7(7)	4.3(5)	-0.9(5)	-1.3(5)
C23	23.5(7)	21.1(7)	29.3(7)	1.9(6)	-5.4(6)	-1.9(6)
C24	16.2(7)	24.6(7)	45.0(9)	5.3(7)	-5.8(6)	-5.8(6)
C25	15.9(7)	27.9(8)	40.2(9)	7.3(6)	3.4(6)	-2.5(6)
C26	15.8(6)	26.2(7)	25.8(7)	4.3(6)	2.2(5)	-0.7(5)
C31	12.3(6)	17.8(6)	21.6(7)	0.9(5)	5.2(5)	-1.4(5)
C32	16.9(6)	19.1(7)	22.9(7)	1.6(5)	3.6(5)	1.8(5)
C33	22.1(7)	28.4(8)	23.4(7)	-3.3(6)	3.9(6)	-2.2(6)
C34	25.3(7)	22.3(7)	35.9(8)	-7.9(6)	12.6(6)	-4.1(6)
C35	20.8(7)	16.7(7)	48.0(9)	2.8(6)	13.5(6)	2.7(5)
C36	15.1(6)	23.7(7)	30.5(8)	8.2(6)	3.2(5)	1.0(5)

Table S5. Bond Lengths for TTG.

Atom	Atom	Length/Å	Atom	Atom	Length/Å
S1	C2	1.7265(13)	C14	C15	1.3973(19)
S1	C5	1.7252(13)	C15	C16	1.3800(19)
O1	C1	1.2154(17)	C21	C22	1.395(2)
N1	C11	1.4191(17)	C21	C26	1.399(2)
N1	C21	1.4145(17)	C22	C23	1.390(2)
N1	C31	1.4318(17)	C23	C24	1.392(2)
C1	C2	1.4484(18)	C24	C25	1.383(2)
C2	C3	1.3753(19)	C25	C26	1.390(2)
C3	C4	1.4033(19)	C31	C32	1.394(2)
C4	C5	1.3815(18)	C31	C36	1.394(2)
C5	C14	1.4659(18)	C32	C33	1.385(2)
C11	C12	1.3937(19)	C33	C34	1.381(2)
C11	C16	1.3998(19)	C34	C35	1.387(2)
C12	C13	1.3868(19)	C35	C36	1.391(2)
C13	C14	1.4018(18)			

Table S6. Bond Angles for TTG.

Atom	Atom	Atom	Angle/°	Atom	Atom	Atom	Angle/°
C5	S1	C2	91.79(6)	C15	C14	C13	117.68(12)
C11	N1	C31	115.72(10)	C16	C15	C14	121.04(12)
C21	N1	C11	122.39(11)	C15	C16	C11	120.99(13)
C21	N1	C31	120.08(11)	C22	C21	N1	120.79(12)
O1	C1	C2	125.35(13)	C22	C21	C26	118.67(13)
C1	C2	S1	121.75(10)	C26	C21	N1	120.54(13)
C3	C2	S1	111.24(10)	C23	C22	C21	120.68(13)
C3	C2	C1	126.98(13)	C22	C23	C24	120.54(14)
C2	C3	C4	112.99(12)	C25	C24	C23	118.77(13)
C5	C4	C3	112.93(12)	C24	C25	C26	121.33(14)
C4	C5	S1	111.05(10)	C25	C26	C21	120.00(14)
C4	C5	C14	127.50(12)	C32	C31	N1	119.13(12)
C14	C5	S1	121.44(10)	C36	C31	N1	121.23(12)
C12	C11	N1	123.27(12)	C36	C31	C32	119.62(13)
C12	C11	C16	118.52(12)	C33	C32	C31	120.04(13)
C16	C11	N1	118.12(12)	C34	C33	C32	120.64(14)
C13	C12	C11	120.23(12)	C33	C34	C35	119.42(14)
C12	C13	C14	121.50(12)	C34	C35	C36	120.71(13)
C13	C14	C5	121.91(12)	C35	C36	C31	119.55(14)
C15	C14	C5	120.40(12)				

Table S7. Torsion Angles for TTG.

A	B	C	D	Angle/°	A	B	C	D	Angle/°
S1	C2	C3	C4	0.77(15)	C12	C11	C16	C15	-1.50(19)
S1	C5	C14	C13	8.73(17)	C12	C13	C14	C5	176.75(11)
S1	C5	C14	C15	-172.46(10)	C12	C13	C14	C15	-2.10(18)
O1	C1	C2	S1	-2.31(19)	C13	C14	C15	C16	1.08(19)
O1	C1	C2	C3	175.61(13)	C14	C15	C16	C11	0.7(2)
N1	C11	C12	C13	-175.93(12)	C16	C11	C12	C13	0.49(18)
N1	C11	C16	C15	175.11(11)	C21	N1	C11	C12	-35.57(19)
N1	C21	C22	C23	178.87(12)	C21	N1	C11	C16	148.00(13)
N1	C21	C26	C25	-179.64(13)	C21	N1	C31	C32	117.49(14)
N1	C31	C32	C33	179.03(12)	C21	N1	C31	C36	-63.90(17)
N1	C31	C36	C35	-179.72(12)	C21	C22	C23	C24	1.2(2)
C1	C2	C3	C4	-177.34(12)	C22	C21	C26	C25	0.8(2)
C2	S1	C5	C4	0.32(10)	C22	C23	C24	C25	0.0(2)
C2	S1	C5	C14	-178.55(10)	C23	C24	C25	C26	-0.8(2)
C2	C3	C4	C5	-0.53(16)	C24	C25	C26	C21	0.4(2)
C3	C4	C5	S1	0.05(14)	C26	C21	C22	C23	-1.6(2)
C3	C4	C5	C14	178.83(12)	C31	N1	C11	C12	129.12(13)
C4	C5	C14	C13	-169.94(12)	C31	N1	C11	C16	-47.31(16)
C4	C5	C14	C15	8.87(19)	C31	N1	C21	C22	167.02(12)
C5	S1	C2	C1	177.60(11)	C31	N1	C21	C26	-12.49(19)
C5	S1	C2	C3	-0.62(10)	C31	C32	C33	C34	0.6(2)
C5	C14	C15	C16	-177.78(11)	C32	C31	C36	C35	-1.1(2)
C11	N1	C21	C22	-28.93(19)	C32	C33	C34	C35	-0.9(2)
C11	N1	C21	C26	151.56(13)	C33	C34	C35	C36	0.2(2)
C11	N1	C31	C32	-47.58(17)	C34	C35	C36	C31	0.8(2)
C11	N1	C31	C36	131.03(13)	C36	C31	C32	C33	0.4(2)
C11	C12	C13	C14	1.33(19)					

Table S8. Hydrogen Atom Coordinates (Å×10^4) and Isotropic Displacement Parameters (Å^2×10^3) for TTG.

Atom	*x*	*y*	*z*	U(eq)
H1	6740	-690	3622	21
H3	6264	2997	3868	21
H4	5193	4739	4013	20
H12	2482	563	4083	21
H13	3591	-292	3885	20
H15	4094	5616	3863	19
H16	2984	6475	4012	21
H22	1741	2028	2617	25
H23	781	193	2013	30

H24	-183	268	2876	35
H25	-169	2193	4347	34
H26	793	3979	4983	27
H32	2582	4217	6084	23
H33	2561	6591	7372	29
H34	1887	9457	7126	33
H35	1250	9990	5563	33
H36	1281	7662	4247	28

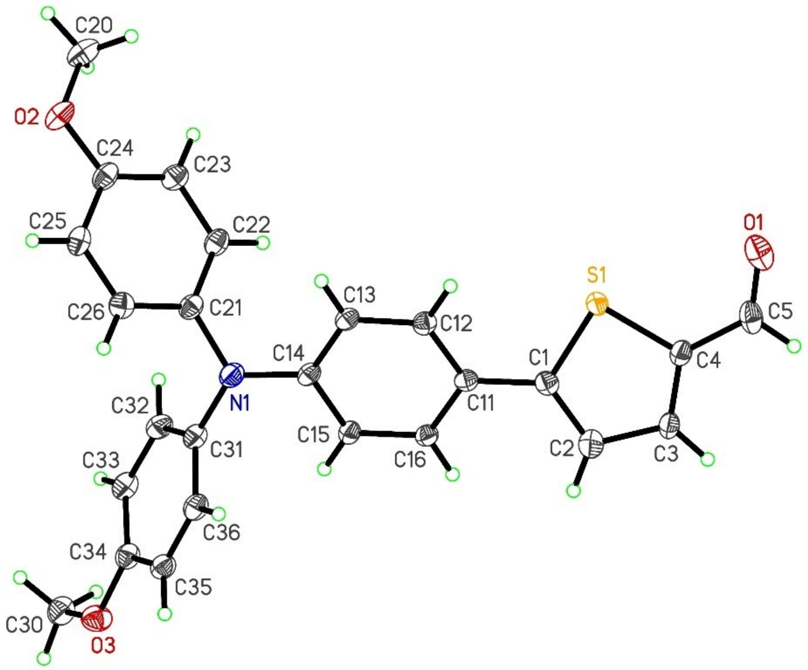

Table S9. Crystal data and structure refinement for TTY.

Identification code	TTY
Empirical formula	$C_{25}H_{21}NO_3S$
Formula weight	415.49
Temperature/K	100.01(10)
Crystal system	monoclinic
Space group	$P2_1/n$
a/Å	9.89163(18)
b/Å	19.5816(3)
c/Å	10.94012(19)
α/°	90
β/°	107.5934(19)
γ/°	90
Volume/Å^3	2019.92(6)
Z	4

ρ_{calc}g/cm^3	1.366
μ/mm^{-1}	1.647
F(000)	872.0
Crystal size/mm^3	0.2 × 0.15 × 0.1
Radiation	CuKα (λ = 1.54184)
2Θ range for data collection/°	9.032 to 134.942
Index ranges	-11 ≤ h ≤ 11, -21 ≤ k ≤ 23, -13 ≤ l ≤ 8
Reflections collected	11028
Independent reflections	3611 [R_{int} = 0.0224, R_{sigma} = 0.0228]
Data/restraints/parameters	3611/240/390
Completeness to theta = 66.5°	99.2%
Goodness-of-fit on F^2	1.007
Final R indexes [I>=2σ (I)]	R_1 = 0.0325, wR_2 = 0.0886
Final R indexes [all data]	R_1 = 0.0363, wR_2 = 0.0919
Largest diff. peak/hole / e Å^{-3}	0.25/-0.33

Table S10. Fractional Atomic Coordinates (×10^4) and Equivalent Isotropic Displacement Parameters (Å^2×10^3) for TTY. U_{eq} is defined as 1/3 of of the trace of the orthogonalised U_{IJ} tensor.

Atom	*x*	*y*	*z*	U(eq)
S1	2189(2)	5267.5(11)	-1358(2)	24.7(4)
S1A	1980(3)	4645.8(14)	509(2)	30.7(5)
O1	-129.5(16)	4621.7(8)	-3601.7(15)	41.6(3)
O1A	-1064(4)	3810(2)	-2289(4)	46.1(8)
O2	9342.1(11)	9190.6(5)	959.7(10)	34.8(2)
O3	8827.3(11)	6876.8(5)	8372.9(9)	34.0(2)
N1	7445.0(12)	6905.6(5)	3030.2(10)	26.9(2)
C1	2918(3)	5194.4(15)	304(2)	23.3(6)
C2	2293(5)	4697(3)	828(5)	34.0(9)
C3	1159(2)	4366.3(9)	-153(2)	25.0(4)
C4	988(2)	4612.9(12)	-1365(3)	24.5(5)
C5	-11(2)	4389.3(10)	-2547(2)	32.5(4)
C11	4079(5)	5634(3)	988(3)	20.9(7)
C12	4897(7)	5992(4)	355(6)	22.9(10)
C13	6035(9)	6409(5)	975(6)	22.1(12)
C14	6351(10)	6491(5)	2309(7)	21.5(11)
C15	5542(6)	6137(3)	2978(5)	22.2(9)
C16	4434(5)	5713(2)	2322(3)	22.1(8)
C1A	2835(8)	5244(4)	-90(7)	28.6(13)
C2A	2170(20)	5257(11)	-1320(20)	26(3)
C3A	1168(6)	4823(3)	-1901(6)	39.8(11)
C4A	865(7)	4449(4)	-979(8)	34.2(12)

C5A	-300(6)	3957(3)	-1224(5)	42.3(10)
C11A	4068(15)	5647(8)	729(10)	28.2(17)
C12A	4994(18)	6007(10)	227(16)	29(2)
C13A	6010(20)	6402(13)	1093(17)	30(3)
C14A	6280(30)	6470(14)	2400(19)	28(3)
C15A	5359(16)	6076(9)	2847(14)	31(2)
C16A	4308(12)	5692(6)	2022(10)	28.2(18)
C20	8322.0(18)	9659.1(7)	209.1(16)	38.6(3)
C21	7886.8(14)	7480.2(6)	2440.6(12)	24.7(3)
C22	6916.0(14)	7904.1(6)	1593.8(13)	26.7(3)
C23	7359.7(14)	8476.7(7)	1073.0(13)	27.6(3)
C24	8798.9(14)	8636.6(7)	1411.4(13)	27.8(3)
C25	9779.6(14)	8215.2(7)	2263.1(13)	28.7(3)
C26	9326.5(14)	7640.5(7)	2768.1(12)	27.8(3)
C30	8592.6(16)	7459.6(7)	9064.7(13)	33.7(3)
C31	7819.0(13)	6918.5(7)	4405.2(12)	25.4(3)
C32	7603.8(14)	7502.1(7)	5039.9(13)	27.6(3)
C33	7941.7(14)	7513.2(7)	6369.9(13)	27.6(3)
C34	8474.1(13)	6928.2(7)	7067.3(13)	26.7(3)
C35	8698.5(14)	6339.3(7)	6434.2(13)	27.3(3)
C36	8388.2(14)	6338.0(7)	5115.8(13)	26.6(3)

Table S11. Anisotropic Displacement Parameters (Å^2×10^3) for TTY. The Anisotropic displacement factor exponent takes the form: $-2\pi^2[h^2a^{*2}U_{11}+2hka^*b^*U_{12}+\ldots]$.

Atom	U_{11}	U_{22}	U_{33}	U_{23}	U_{13}	U_{12}
S1	25.1(6)	21.7(6)	23.4(3)	0.7(3)	1.3(3)	-2.2(6)
S1A	32.8(11)	29.8(8)	31.2(10)	3.1(7)	12.3(8)	-0.1(7)
O1	35.6(7)	43.8(8)	38.5(7)	-10.0(6)	0.9(6)	4.2(6)
O1A	43.0(14)	43.2(15)	48.2(13)	-2.8(11)	7.7(11)	-0.9(12)
O2	37.6(5)	23.2(5)	48.6(6)	3.0(4)	20.5(5)	-4.9(4)
O3	44.9(6)	31.2(5)	27.4(5)	-0.3(4)	13.2(4)	1.8(4)
N1	31.4(6)	22.7(5)	27.7(6)	0.0(4)	10.4(5)	-4.1(4)
C1	23.0(9)	20.4(10)	24.9(10)	-0.9(8)	4.7(8)	3.8(7)
C2	32.4(17)	32.0(13)	35.0(15)	-1.4(11)	6.2(11)	-4.9(11)
C3	21.4(9)	21.4(8)	30.2(9)	-3.1(7)	4.9(7)	-3.4(7)
C4	22.2(9)	20.1(11)	30.7(10)	-1.5(8)	7.1(8)	-1.8(7)
C5	24.8(9)	27.7(9)	39.8(8)	-10.3(7)	1.8(7)	1.9(7)
C11	21.9(10)	18.2(12)	23.0(14)	1.3(10)	7.4(10)	3.0(8)
C12	25.0(14)	19.3(16)	24.6(14)	-0.7(10)	7.8(10)	1.1(12)
C13	24.6(16)	16.9(19)	26.3(15)	1.3(11)	9.6(11)	0.7(15)
C14	22.0(17)	17(2)	27.8(16)	1.1(11)	10.4(10)	0.5(15)
C15	22.5(15)	19.2(16)	24.8(13)	-0.7(9)	7.2(10)	-1.6(12)

C16	24.3(13)	21.7(13)	21.0(13)	1.2(10)	8(1)	-1.8(10)
C1A	30(2)	23(2)	36(2)	2.8(16)	14.5(16)	3.4(16)
C2A	25(4)	23(5)	34(2)	0.6(18)	14.7(18)	13(4)
C3A	40(2)	39(2)	40.6(18)	0.2(15)	11.1(15)	-0.2(17)
C4A	33.4(18)	31(2)	37.9(18)	-0.6(15)	10.7(15)	3.6(16)
C5A	38.8(18)	39(2)	46.8(17)	0.3(14)	9.5(13)	-2.5(16)
C11A	31(2)	22(3)	34(3)	5(2)	14.7(19)	3(2)
C12A	30(3)	22(4)	37(3)	6(2)	16(2)	2(3)
C13A	31(4)	25(5)	38(3)	4(2)	17(2)	0(4)
C14A	28(4)	22(5)	37(3)	4(2)	15(2)	3(4)
C15A	32(4)	28(4)	36(3)	3(2)	16(2)	-3(3)
C16A	30(3)	23(3)	36(3)	4(2)	15(2)	-1(2)
C20	48.2(9)	25.0(7)	48.7(9)	4.8(6)	23.5(7)	-2.7(6)
C21	28.9(6)	20.8(6)	27.1(6)	-2.6(5)	12.5(5)	-2.6(5)
C22	25.2(6)	23.0(6)	34.4(7)	-1.8(5)	13.0(5)	-1.1(5)
C23	29.3(7)	22.0(6)	33.6(7)	0.7(5)	12.7(5)	2.1(5)
C24	33.6(7)	20.7(6)	34.4(7)	-4.8(5)	17.9(6)	-4.0(5)
C25	25.3(6)	28.0(7)	34.6(7)	-4.9(5)	12.0(5)	-4.8(5)
C26	28.2(7)	25.7(6)	29.9(6)	-1.8(5)	9.3(5)	0.8(5)
C30	40.9(8)	32.4(7)	30.7(7)	-5.5(5)	15.0(6)	-5.8(6)
C31	25.7(6)	24.4(6)	27.9(6)	0.2(5)	10.8(5)	-2.9(5)
C32	28.4(6)	22.3(6)	33.8(7)	3.2(5)	12.0(5)	1.5(5)
C33	29.9(7)	22.9(6)	33.7(7)	-3.6(5)	15.0(5)	-0.5(5)
C34	25.6(6)	27.7(7)	28.9(6)	-0.6(5)	11.3(5)	-3.0(5)
C35	26.7(6)	22.9(6)	32.4(7)	2.7(5)	9.2(5)	0.0(5)
C36	26.9(6)	21.9(6)	32.8(7)	-2.4(5)	11.7(5)	-0.7(5)

Table S12. Bond Lengths for TTY.

Atom	Atom	Length/Å	Atom	Atom	Length/Å
S1	C1	1.747(3)	C15	C16	1.391(4)
S1	C4	1.746(3)	C1A	C2A	1.31(2)
S1A	C1A	1.690(7)	C1A	C11A	1.501(13)
S1A	C4A	1.712(7)	C2A	C3A	1.32(2)
O1	C5	1.213(3)	C3A	C4A	1.351(9)
O1A	C5A	1.216(7)	C4A	C5A	1.464(9)
O2	C20	1.4259(19)	C11A	C12A	1.394(10)
O2	C24	1.3676(16)	C11A	C16A	1.365(9)
O3	C30	1.4266(17)	C12A	C13A	1.392(14)
O3	C34	1.3677(16)	C13A	C14A	1.379(13)
N1	C14	1.392(9)	C14A	C15A	1.394(13)
N1	C14A	1.43(2)	C15A	C16A	1.376(10)
N1	C21	1.4297(17)	C21	C22	1.3898(19)

N1	C31	1.4366(17)	C21	C26	1.3952(19)
C1	C2	1.369(6)	C22	C23	1.3876(18)
C1	C11	1.449(4)	C23	C24	1.3940(19)
C2	C3	1.450(4)	C24	C25	1.394(2)
C3	C4	1.373(4)	C25	C26	1.3864(19)
C4	C5	1.438(3)	C31	C32	1.3867(18)
C11	C12	1.402(4)	C31	C36	1.3961(19)
C11	C16	1.403(3)	C32	C33	1.3911(19)
C12	C13	1.390(6)	C33	C34	1.3888(19)
C13	C14	1.406(5)	C34	C35	1.3971(19)
C14	C15	1.417(5)	C35	C36	1.3810(19)

Table S13. Bond Angles for TTY.

Atom	Atom	Atom	Angle/°	Atom	Atom	Atom	Angle/°
C4	S1	C1	90.60(18)	C3A	C4A	C5A	124.1(6)
C1A	S1A	C4A	92.4(4)	C5A	C4A	S1A	124.6(6)
C24	O2	C20	115.56(11)	O1A	C5A	C4A	123.9(6)
C34	O3	C30	117.64(11)	C12A	C11A	C1A	122.9(8)
C14	N1	C21	119.6(3)	C16A	C11A	C1A	120.6(8)
C14	N1	C31	121.0(2)	C16A	C11A	C12A	116.5(10)
C14A	N1	C21	124.2(8)	C13A	C12A	C11A	116.1(15)
C14A	N1	C31	115.5(6)	C14A	C13A	C12A	129(2)
C21	N1	C31	116.19(10)	C13A	C14A	N1	115.7(12)
C2	C1	S1	113.5(2)	C13A	C14A	C15A	112(2)
C2	C1	C11	126.4(3)	C15A	C14A	N1	132.7(15)
C11	C1	S1	120.1(2)	C16A	C15A	C14A	121.2(15)
C1	C2	C3	110.7(3)	C11A	C16A	C15A	125.2(10)
C4	C3	C2	113.6(3)	C22	C21	N1	121.80(11)
C3	C4	S1	111.67(17)	C22	C21	C26	118.87(12)
C3	C4	C5	127.7(2)	C26	C21	N1	119.26(12)
C5	C4	S1	120.6(2)	C23	C22	C21	121.05(12)
O1	C5	C4	125.7(2)	C22	C23	C24	119.79(13)
C12	C11	C1	121.8(3)	O2	C24	C23	124.30(13)
C12	C11	C16	117.3(3)	O2	C24	C25	116.17(12)
C16	C11	C1	120.8(3)	C23	C24	C25	119.54(12)
C13	C12	C11	123.8(6)	C26	C25	C24	120.22(12)
C12	C13	C14	117.8(8)	C25	C26	C21	120.53(12)
N1	C14	C13	123.1(4)	C32	C31	N1	120.60(11)
N1	C14	C15	117.1(5)	C32	C31	C36	119.29(12)
C13	C14	C15	119.8(7)	C36	C31	N1	120.10(11)
C16	C15	C14	120.4(5)	C31	C32	C33	120.79(12)
C15	C16	C11	120.9(3)	C34	C33	C32	119.54(12)

C2A	C1A	S1A	105.0(12)	O3	C34	C33	124.70(12)
C2A	C1A	C11A	132.0(13)	O3	C34	C35	115.35(12)
C11A	C1A	S1A	123.0(6)	C33	C34	C35	119.94(12)
C1A	C2A	C3A	124(2)	C36	C35	C34	120.06(12)
C2A	C3A	C4A	107.2(13)	C35	C36	C31	120.34(12)
C3A	C4A	S1A	111.3(5)				

Table S14. Torsion Angles for TTY.

A	B	C	D	Angle/°	A	B	C	D	Angle/°
S1	C1	C2	C3	-0.9(5)	C2A	C3A	C4A	S1A	-4.6(11)
S1	C1	C11	C12	-17.2(6)	C2A	C3A	C4A	C5A	173.4(9)
S1	C1	C11	C16	163.2(3)	C3A	C4A	C5A	O1A	4.0(10)
S1	C4	C5	O1	-0.7(3)	C4A	S1A	C1A	C2A	2.5(10)
S1A	C1A	C2A	C3A	-6.7(18)	C4A	S1A	C1A	C11A	-178.2(9)
S1A	C1A	C11A	C12A	163.2(11)	C11A	C1A	C2A	C3A	174.2(12)
S1A	C1A	C11A	C16A	-18.8(15)	C11A	C12A	C13A	C14A	3(4)
S1A	C4A	C5A	O1A	-178.2(5)	C12A	C11A	C16A	C15A	1.8(18)
O2	C24	C25	C26	-179.83(11)	C12A	C13A	C14A	N1	178(3)
O3	C34	C35	C36	-179.46(11)	C12A	C13A	C14A	C15A	0(4)
N1	C14	C15	C16	-179.9(6)	C13A	C14A	C15A	C16A	-1(3)
N1	C14A	C15A	C16A	-180(2)	C14A	N1	C21	C22	37.9(13)
N1	C21	C22	C23	177.18(11)	C14A	N1	C21	C26	-145.2(13)
N1	C21	C26	C25	-176.61(11)	C14A	N1	C31	C32	-112.0(13)
N1	C31	C32	C33	178.88(11)	C14A	N1	C31	C36	67.2(13)
N1	C31	C36	C35	-177.49(11)	C14A	C15A	C16A	C11A	1(2)
C1	S1	C4	C3	0.2(2)	C16A	C11A	C12A	C13A	-3(2)
C1	S1	C4	C5	-179.2(2)	C20	O2	C24	C23	7.01(18)
C1	C2	C3	C4	1.1(5)	C20	O2	C24	C25	-173.05(12)
C1	C11	C12	C13	-178.9(9)	C21	N1	C14	C13	27.5(11)
C1	C11	C16	C15	-179.6(4)	C21	N1	C14	C15	-153.0(6)
C2	C1	C11	C12	162.2(5)	C21	N1	C14A	C13A	30(3)
C2	C1	C11	C16	-17.4(6)	C21	N1	C14A	C15A	-152(2)
C2	C3	C4	S1	-0.8(3)	C21	N1	C31	C32	46.34(16)
C2	C3	C4	C5	178.6(3)	C21	N1	C31	C36	-134.48(12)
C3	C4	C5	O1	179.9(2)	C21	C22	C23	C24	-0.73(19)
C4	S1	C1	C2	0.4(4)	C22	C21	C26	C25	0.38(19)
C4	S1	C1	C11	179.9(3)	C22	C23	C24	O2	-179.53(12)
C11	C1	C2	C3	179.6(4)	C22	C23	C24	C25	0.54(19)
C11	C12	C13	C14	-2.0(13)	C23	C24	C25	C26	0.10(19)
C12	C11	C16	C15	0.8(6)	C24	C25	C26	C21	-0.57(19)
C12	C13	C14	N1	-178.7(10)	C26	C21	C22	C23	0.27(19)

C12	C13	C14	C15	1.8(12)	C30	O3	C34	C33	1.95(19)
C13	C14	C15	C16	-0.3(11)	C30	O3	C34	C35	-178.79(12)
C14	N1	C21	C22	41.6(5)	C31	N1	C14	C13	-173.6(7)
C14	N1	C21	C26	-141.5(5)	C31	N1	C14	C15	6.0(10)
C14	N1	C31	C32	-113.3(6)	C31	N1	C14A	C13A	-173.6(16)
C14	N1	C31	C36	65.9(6)	C31	N1	C14A	C15A	5(3)
C14	C15	C16	C11	-1.0(8)	C31	N1	C21	C22	-118.36(13)
C16	C11	C12	C13	0.7(10)	C31	N1	C21	C26	58.55(16)
C1A	S1A	C4A	C3A	1.3(6)	C31	C32	C33	C34	-1.31(19)
C1A	S1A	C4A	C5A	-176.7(6)	C32	C31	C36	C35	1.70(19)
C1A	C2A	C3A	C4A	7.7(19)	C32	C33	C34	O3	-179.23(12)
C1A	C11A	C12A	C13A	175(2)	C32	C33	C34	C35	1.54(19)
C1A	C11A	C16A	C15A	-176.3(12)	C33	C34	C35	C36	-0.16(19)
C2A	C1A	C11A	C12A	-18(2)	C34	C35	C36	C31	-1.47(19)
C2A	C1A	C11A	C16A	160.1(14)	C36	C31	C32	C33	-0.30(19)

Table S15. Hydrogen Atom Coordinates (Å×10^4) and Isotropic Displacement Parameters (Å^2×10^3) for TTY.

Atom	*x*	*y*	*z*	U(eq)
H2	2560	4586	1695	41
H3	600	4020	22	30
H5	-619	4035	-2498	39
H12	4666	5947	-531	27
H13	6569	6627	522	27
H15	5755	6190	3861	27
H16	3920	5478	2774	27
H2A	2424	5592	-1815	31
H3A	746	4780	-2781	48
H5A	-467	3744	-524	51
H12A	4934	5985	-637	34
H13A	6597	6657	740	36
H15A	5453	6073	3719	37
H16A	3715	5444	2374	34
H20A	7725	9429	-534	58
H20B	8802	10031	-55	58
H20C	7753	9832	711	58
H22	5954	7802	1373	32
H23	6699	8753	500	33
H25	10741	8320	2493	34
H26	9988	7360	3330	33
H30A	7610	7586	8761	51
H30B	9165	7833	8936	51

H30C	8845	7352	9962	51
H32	7229	7891	4571	33
H33	7812	7910	6789	33
H35	9057	5948	6901	33
H36	8559	5948	4699	32

Table S16. Atomic Occupancy for TTY.

**Atom**	***Occupancy***	**Atom**	***Occupancy***	**Atom**	***Occupancy***
S1	0.7	S1A	0.3	O1	0.7
O1A	0.3	C1	0.7	C2	0.7
H2	0.7	C3	0.7	H3	0.7
C4	0.7	C5	0.7	H5	0.7
C11	0.7	C12	0.7	H12	0.7
C13	0.7	H13	0.7	C14	0.7
C15	0.7	H15	0.7	C16	0.7
H16	0.7	C1A	0.3	C2A	0.3
H2A	0.3	C3A	0.3	H3A	0.3
C4A	0.3	C5A	0.3	H5A	0.3
C11A	0.3	C12A	0.3	H12A	0.3
C13A	0.3	H13A	0.3	C14A	0.3
C15A	0.3	H15A	0.3	C16A	0.3
H16A	0.3				

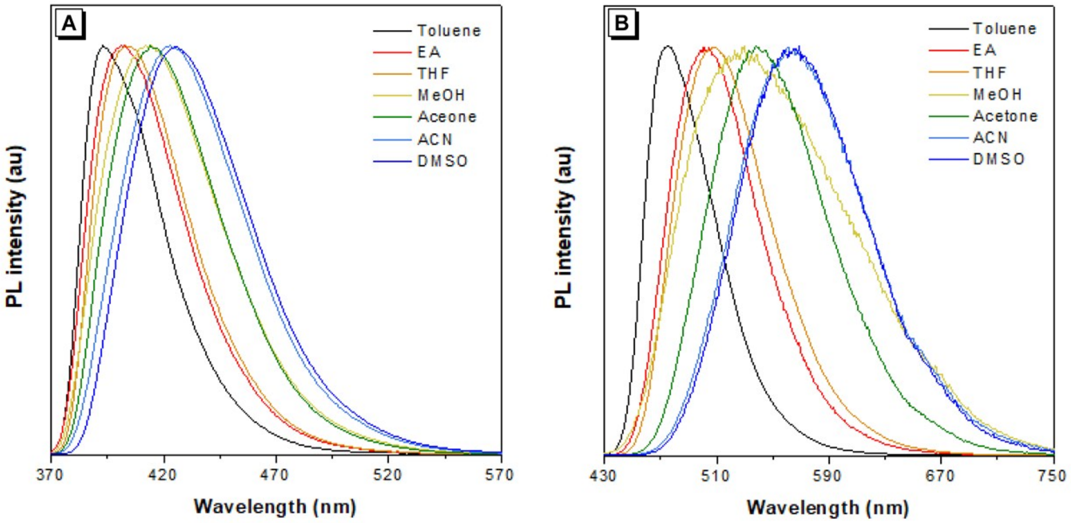

Figure S3. PL spectra of A) TTV and B) TTG in different solvents for solvatochromic effect evaluation.

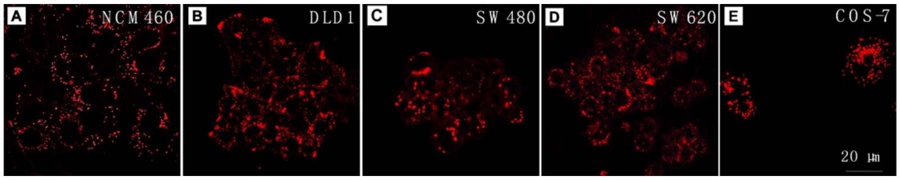

Figure S4. Extension of the LDs-specific imaging strategy using TTNIR to other cells. Confocal images of living A) NCM460, B) DLD1, C) SW480, D) SW620 and E) COS-7 cells after incubation with TTNIR (1 μM) for 20 min. λ_{ex}: 488 nm.

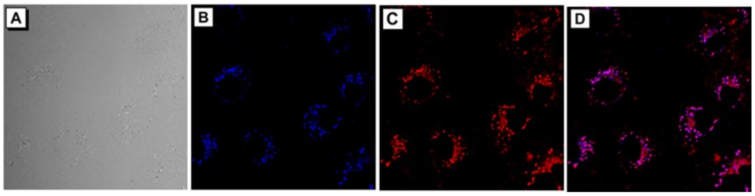

Figure S5. Colocalization imaging of COS-7 cells stained with TTV and Nile Red. A) Bright-field and B) confocal images of COS-7 cells stained with TTV and C) Nile Red. D) Merged images of panels (B) and (C). TTV is excited with a 405 nm laser (14 % laser power), and the emission is collected with the 415-550 nm filter. Nile Red is excited with a 514 nm laser (6.5% laser power), and the emission is collected with the 580-620 nm filter. Concentrations: TTV (5 μM), Nile Red (5 μM). Pearson's correlation coefficient: 0.90

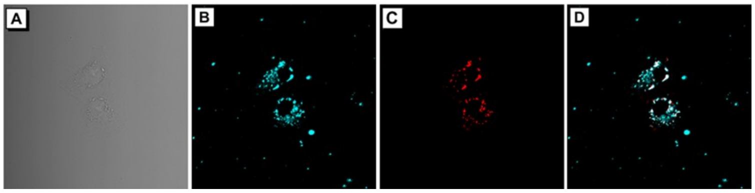

Figure S6. Colocalization imaging of COS-7 cells stained with TTB and Nile Red. A) Bright-field and B) confocal images of COS-7 cells stained with TTB and C) Nile Red. D) Merged images of panels (B) and (C). TTB is excited with a 405 nm laser (40 % laser power), and the emission is collected with the 415-550 nm filter. Nile Red is excited with a 514 nm laser (6.5% laser power), and the emission is collected with the 580-620 nm filter. Concentrations: TTB (5 μM), Nile Red (5 μM). Pearson's correlation coefficient: 0.91

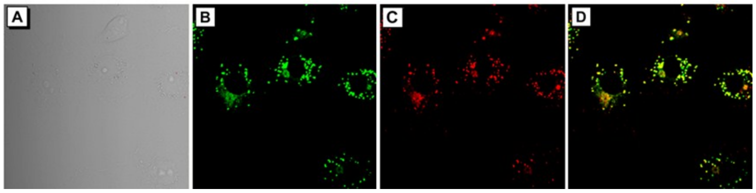

Figure S7. Colocalization imaging of COS-7 cells stained with TTG and Nile Red. A) Bright-field and B) confocal images of COS-7 cells stained with TTG and C) Nile Red. D) Merged images of panels (B) and (C). TTG is excited with a 405 nm laser (0.2% laser power), and the emission is collected with the 480-545 nm filter. Nile Red is excited with a 514 nm laser (6.5% laser power), and the emission is collected with the 580-630 nm filter. Concentrations: TTG (5 μM), Nile Red (5 μM). Pearson's correlation coefficient: 0.90

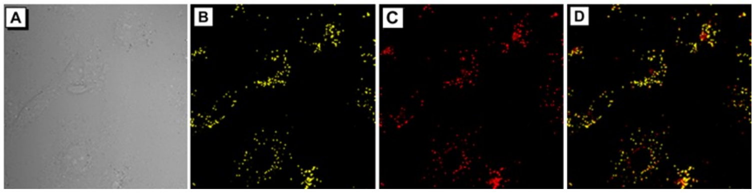

Figure S8. Colocalization imaging of COS-7 cells stained with TTY and Nile Red. A) Bright-field and B) confocal images of COS-7 cells stained with TTY and C) Nile Red. D) Merged images of panels (B) and (C) and

(C). TTY is excited with a 405 nm laser (0.2% laser power), and the emission is collected with the 490-625 nm filter. Nile Red is excited with a 514 nm laser (6.5% laser power), and the emission is collected with the 580-630 nm filter. Concentrations: TTY (5 μM), Nile Red (5 μM). Pearson's correlation coefficient: 0.92

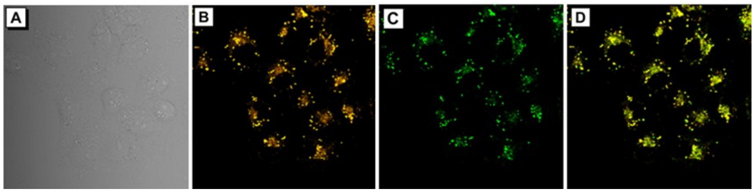

Figure S9. Colocalization imaging of COS-7 cells stained with TTO and BODIPY493/503 Green. A) Bright-field and B) confocal images of COS-7 cells stained with TTO and C) BODIPY493/503 Green. D) Merged images of panels (B) and (C). TTR is excited with a 488 nm laser (8% laser power), and the emission is collected with the 560-650 nm filter. BODIPY493/503 Green is excited with a 488 nm laser (2.8% laser power), and the emission is collected with the 500-540 nm filter. Concentrations: TTO (5 μM), BODIPY493/503 Green (5 μM). Pearson's correlation coefficient: 0.95

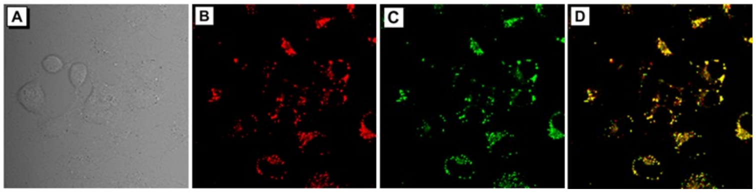

Figure S10. Colocalization imaging of COS-7 cells stained with TTR and BODIPY493/503 Green. A) Bright-field and B) confocal images of COS-7 cells stained with TTR and C) BODIPY493/503 Green. D) Merged images of panels (B) and (C). TTR is excited with a 488 nm laser (8% laser power), and the emission is collected with the 560-740 nm filter. BODIPY493/503 Green is excited with a 488 nm laser (2.8% laser power), and the emission is collected with the 500-540 nm filter. Concentrations: TTR (5 μM), BODIPY493/503 Green (5 μM). Pearson's correlation coefficient: 0.94

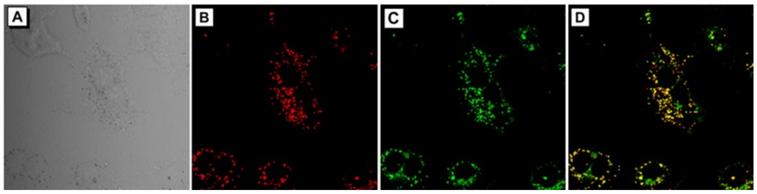

Figure S11. Colocalization imaging of COS-7 cells stained with TTDR and BODIPY493/503 Green. A) Bright-field and B) confocal images of COS-7 cells stained with TTDR and C) BODIPY493/503 Green. D) Merged images of panels (B) and (C). TTDR is excited with a 488 nm laser (0.1% laser power), and the emission is collected with the 570-740 nm filter. BODIPY493/503 Green is excited with a 488 nm laser (2.8% laser power), and the emission is collected with the 500-540 nm filter. Concentrations: TTDR (5 μM), BODIPY493/503 Green (5 μM). Pearson's correlation coefficient: 0.93

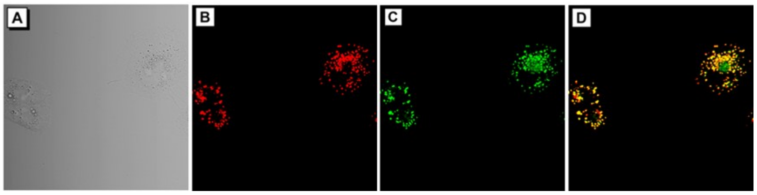

Figure S12. Colocalization imaging of COS-7 cells stained with TTNIR and BODIPY493/503 Green. A) Bright-field and B) confocal images of COS-7 cells stained with TTNIR and C) BODIPY493/503 Green. D) Merged images of panels (B) and (C). TTNIR is excited with a 560 nm laser (0.1% laser power), and the emission is collected with the 570-740 nm filter. BODIPY493/503 Green is excited with a 488 nm laser (2.8% laser power), and the emission is collected with the 500-540 nm filter. Concentrations: TTNIR (5 μM), BODIPY493/503 Green (5 μM). Pearson's correlation coefficient: 0.90

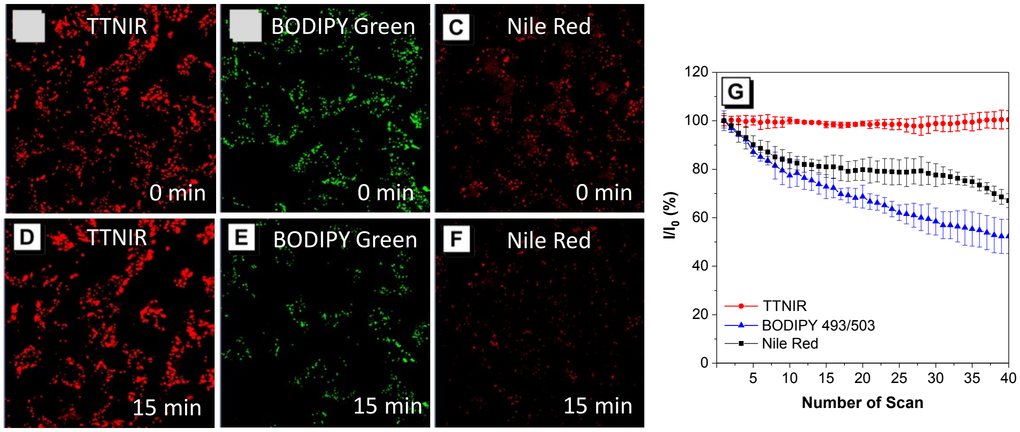

Figure S13. Photostability investigation of TTNIR compared with BODIPY493/503 Green and Nile Red. Confocal images of Hela cells A,B,C) before (0 min) and D,E,F) after the laser irradiation for 15 min stained with A,D) TTNIR, B,E) BODIPY493/503 Green, and C,F) Nile Red. G) Loss in fluorescence of Hela cells stained with TTNIR, BODIPY493/503 Green and Nile Red with the number of scans of laser irradiation. Concentrations: TTNIR (1 μM), BODIPY493/503 Green (500 nM), Nile Red (500 nM). Laser power: 1%. Scanning rate: 22.4 s per frame. Scale bar = 20 μm.

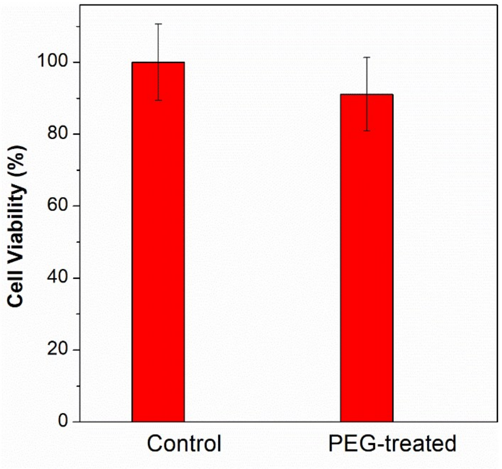

Figure S14. MTT study of cell viability treated with 50% PEG
